# Spatial propagation of temperate phages within and among biofilms

**DOI:** 10.1073/pnas.2417058122

**Published:** 2025-02-04

**Authors:** James B. Winans, Lanying Zeng, Carey D. Nadell

**Affiliations:** ^a^Department of Biological Sciences, Dartmouth, Hanover, NH 03755; ^b^Department of Microbiology and Immunology, Geisel School of Medicine at Dartmouth, Hanover, NH 03755; ^c^Department of Biochemistry and Biophysics, Center for Phage Technology, Texas A&M University, College Station, TX 77843

**Keywords:** biofilm, phage, matrix, dispersal, spatial ecology

## Abstract

Bacteria form groups composed of cells and a secreted polymeric matrix that controls their spatial organization. These groups—termed biofilms—can act as refuges from environmental disturbances and from biotic threats, including phages. Despite the ubiquity of temperate phages and bacterial biofilms, live propagation of temperate phages within biofilms has not been characterized on cellular spatial scales. Here, we leverage several approaches to track temperate phages and distinguish between lytic and lysogenic host infections. We determine that lysogeny within *Escherichia coli* biofilms initially occurs within a predictable region of cell group packing architecture on the biofilm periphery. Because lysogens are generally found on the periphery of large cell groups, where lytic viral infections also reduce local biofilm structural integrity, lysogens are predisposed to disperse into the passing liquid and are overrepresented in downstream biofilms formed from the dispersal pool of the original biofilm-phage system. Comparing our results with those for virulent phages reveals that temperate phages have unique advantages in propagating over long spatial and time scales within and among bacterial biofilms.

## Significance

Bacteria reside in surface-bound communities, or biofilms, in which they produce extracellular polymers that control their organization in space and time. One of the primary functions of biofilm formation is protection from threats such as viruses (phages), but the microscale dynamics of temperate phage propagation in biofilms is virtually unexplored. Here, we leverage customized tools to study how temperate phages spread through biofilms, how they differ from purely lytic phages, and what these differences might mean for the relative advantages and disadvantages of distinct phage life history strategies specific to the biofilm context.

Across large ranges in spatial scale and phylogenetic distance, many species—from microbes to the largest metazoans—form groups as an adaptive strategy to navigate environmental challenges (1–5). Microbial collectives, often termed biofilms, are produced either in a free-floating state or attached to surfaces. Biofilms are primarily composed of cells and a suite of secreted polymers, called the extracellular matrix (6–8). This mode of group formation among bacteria is ubiquitous in both natural and human-made environments, and a primary function of collective growth is protection against invading competitors, diffusible antimicrobial compounds, predatory bacteria, and phages (9–11). The microscopic scale dynamics of group protection against these threats are not well known, particularly for phages and predatory bacteria (12–15). Complementing prior work with microscopic observation of phage–biofilm interactions is essential to provide insight into the driving mechanisms of these interactions at the spatial scale on which microbial phenotypes directly manifest.

Microbial predators have a broad diversity of life history patterns. Obligate lytic phages, also called virulent phages, inject their genome into host cells, where the cellular machinery is coopted for phage replication preceding host cell lysis (16). Temperate phages, by contrast, have two different modes of propagation: 1) they may proceed with a lytic infection like virulent phages, killing the host cell and producing a new burst of phage virions, or 2) they may integrate into the bacterial genome or persist as an episome to generate a host lysogen that carries the phage’s genome along with it as it undergoes normal growth and division (17). Once integrated into a host’s genome, prophages can introduce new genes that substantially alter cell physiology, including antibiotic resistance, metabolic capacity, and virulence (18–21). Crucially, lysogens also exhibit superinfection exclusion: They become immune to phages that are the same or closely related to that which lysogenized them. The movement of temperate phages into and out of host genomes often mediates horizontal gene transfer (22); has powerful, ancient, and widespread effects on microbial evolution (23, 24); and can drive lysogenic conversion of pathogens that present major challenges to human health (25). An enormous wealth of knowledge of phage ecology, evolution, and molecular genetics has been gathered over a century of research (16, 26–30). However, despite the ubiquity of biofilms and phages in microbial ecology, we are still learning the fundamentals of how phages interact with biofilm-dwelling hosts. Temperate phage propagation, in particular, remains uncharacterized at cellular and multicellular spatial scales among host bacteria in biofilms.

Recent work has begun to clarify the fundamentals of lytic phage propagation at high spatial and temporal resolution in the biofilm context. This research has shown that, for several species, bacteria within biofilms are often protected from lytic phage exposure (31–35), and that this protection is dependent on biofilm growth and architectural maturity at the time of phage introduction. When *Escherichia coli* is growing in a biofilm context, for example, the hosts’ interactions with lytic phages are profoundly different relative to shaken liquid culture conditions (12, 31, 36). Mature *E. coli* biofilms can maintain net-positive growth in the presence of T7 phages by blocking phage diffusion in a manner dependent on the secretion of curli amyloid fibers (31), a core proteinaceous component of the *E. coli* biofilm matrix. Phages can be trapped within the networks of highly packed cells and curli fiber protein on the periphery of the biofilm, but the phages remain viable and can infect susceptible bacteria attempting to colonize the biofilm exterior from the planktonic phase (36). The spatial constraint on phage and cell movement within biofilms inherently tends to create negative frequency-dependent selection for genetic phage resistance—that is, resistant mutants are selectively favored when rare, but disfavored when common. This result occurs because genetically resistant cells, once they are common, further reduce phage mobility in the system and can prevent susceptible cells from being exposed and infected (37, 38).

The work summarized above has established an early picture of how obligate lytic phages interact with biofilm-dwelling hosts at microscopic spatial scales. In contrast, there is very little analogous work on how temperate phages propagate through biofilm communities, nor has prior research addressed how the patterns of phage propagation at the community scale are driven by cellular scale interaction dynamics. Some previous research has used traditional macroscopic measurements to explore how temperate phage exposure influences biofilms (39). A recent study showed that nonspecific genome insertion by prophages can accelerate bacterial evolution by increasing host mutation rate in biofilms of *P. aeruginosa* (40). Other work with lambdoid phage H-19B and *E. coli* MG1655 indicated that almost all host cells are quickly lysogenized in biofilm populations (41). This work set a precedent that temperate phage exposure in biofilms can be important; but efforts thus far have used techniques that disrupt the physical architecture of the biofilms and average over the entire host and phage populations. Crucial questions therefore remain: How do temperate phages propagate through biofilms at the cellular scale; how do these fine-scale patterns influence population and community structure and composition; and how are the answers to these questions distinct for temperate phages relative to obligate lytic phages (22, 42–44)?

To begin answering these questions, we developed a model system in which host bacteria can be visualized at single-cell resolution, and in which naïve (uninfected) cells, lysogenized cells, ongoing lytic infections, and phage virions can be distinguished by fluorescence microscopy. The model comprises monoculture biofilms of *E. coli* AR3110 cultivated in microfluidic devices under constant media flow, to which temperate phage λ (or, in selected control experiments, obligate lytic phage T7 or λΔ*cI*) can be added at any time during or after host cell surface colonization, cell growth, and biofilm matrix secretion. *E. coli* is among the most extensively studied organisms in microbiology, and its mechanisms of biofilm formation have likewise been dissected thoroughly (45–52). Similarly, temperate phage λ has been studied in detail over many decades of work (53–63). Here, we assess how this phage and its host interact within biofilms at the resolution of individual cells and phage virions. Our study yields insight into the principles of temperate phage propagation at cellular and multicellular biofilm scales, the impact of the temperate phage life history strategy on host biofilm architecture, and the nature of local versus distal dispersal-based spread of temperate phages versus virulent phages within and among biofilms.

## Results

*E. coli* AR3110 was engineered to constitutively produce the far-red fluorescent protein mKate2. AR3110 is a K-12 derivative with biofilm matrix production restored relative to the parental W3110 K-12 lineage (45). The resulting strain was used to inoculate microfluidic devices composed of glass coverslips bonded to polydimethylsiloxane molds; chambers measured 5,000 μm long, 500 μm wide, and 70 μm in height from glass substratum to PDMS ceiling (*SI Appendix*, Fig. S1). After a 45-min period of stationary conditions to allow cells to attach to the glass, M9 minimal media with 0.5% maltose was introduced at a flow rate of 0.1 μL min^−1^ (average flow velocity = 45 μm/s) for 48 h. We chose maltose as the sole carbon source to ensure that cells produce the LamB maltoporin, which is the attachment site for phage λ (64). The media influent was then switched to a new syringe with the same growth media plus λ phages at a concentration of 10^4^ PFU μL^−1^ for 24 h. This strain of λ, λLZ1367, has a copy of the teal fluorescent protein mTurquoise2 translationally fused to λD, which encodes the capsid protein gpD (65, 66). λLZ1367 also contains a downstream transcriptional fusion of *mKO2* to the *cI* locus encoding Repressor, which causes lysogenized hosts to produce the orange fluorescent protein mKO2 (66–68). Overall, this arrangement of constitutive and regulated fluorescent protein constructs on the bacterial and phage genomes allows for high-resolution spatial tracking of nonlysogenized hosts (mKate2), lysogenized hosts (mKO2), lytic hosts, and λ phage virions (mTurquoise2). By comparing total fluorescence of serial dilutions of mTurquoise2-labeled phage virions to PFU titers, we confirmed that the teal fluorescence intensity of λLZ1367 scales linearly with phage titer in our image data (*SI Appendix*, Fig. S2*B*). Furthermore, the production of mKO2 allows us to accurately measure the fraction of lysogenized cells for all experiments below, which we confirmed by comparing lysogen quantification via mKO2 fluorescence thresholding to measurements of lysogen CFU counts within control chambers (*SI Appendix*, Fig. S2 *A* and *C*).

### Lytic Infections and Host Lysogenization are Restricted to the Biofilm Periphery in a Manner Dependent on Biofilm Matrix and Cell Packing.

**Fig. 1.**
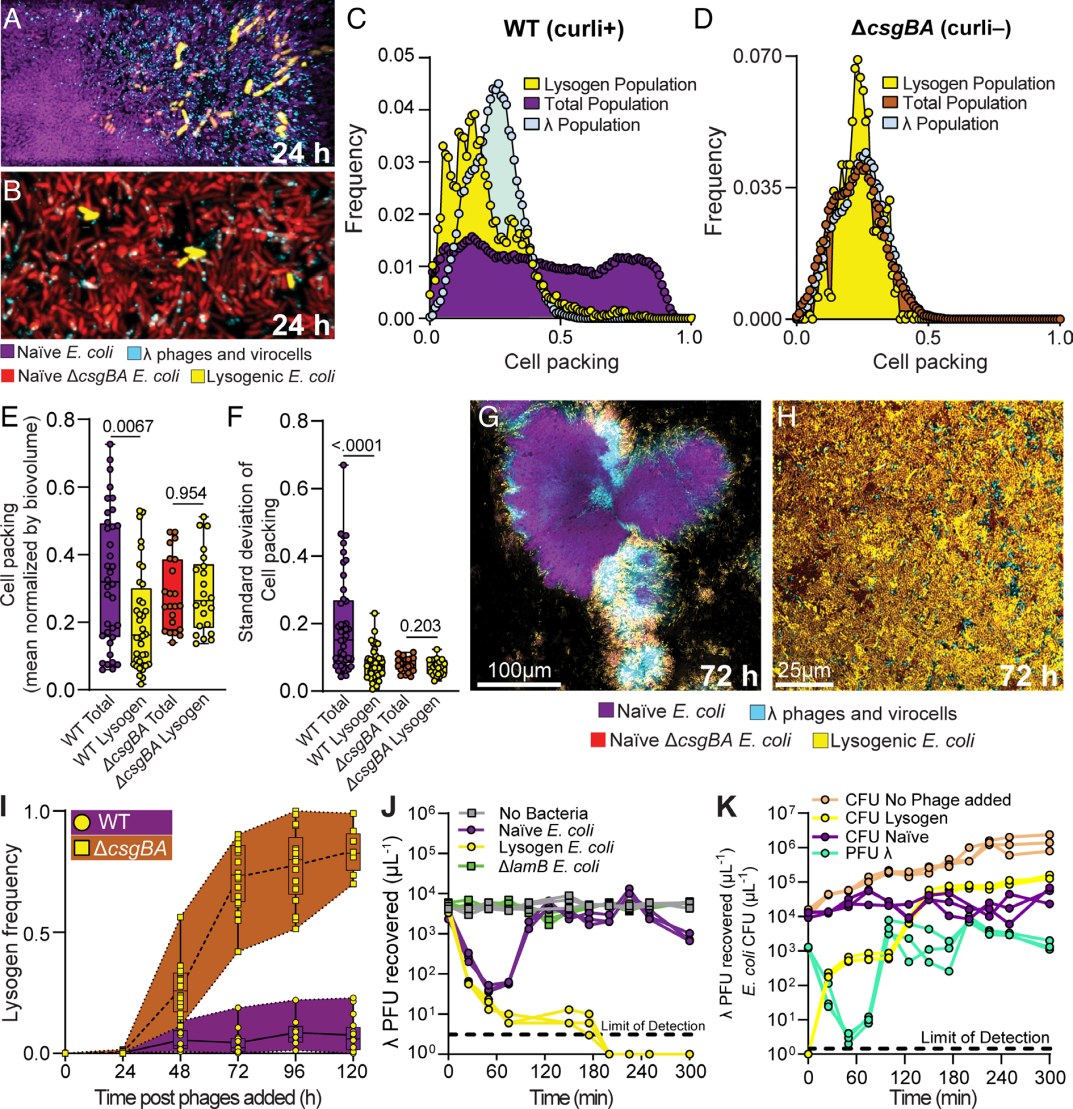
*λ* phages and their lysogenized hosts are mostly restricted to the outer periphery of biofilm cell groups, which is stable over days of growth; this pattern is closely dependent on host biofilm architecture. (*A*) WT *E. coli* biofilms (purple) invaded with λ phages (turquoise) for 24 h, with some cells becoming lysogenized (yellow). (*B*) Δ*csgBA* biofilms (red) invaded with λ phages (turquoise) for 24 h, with some cells becoming lysogenized (yellow). Images in (*A*) and (*B*) are 3-dimensional renderings (for WT: 64Lx32Wx30H μm; for Δ*csgBA*: 64Lx32Wx8H μm). (*C* and *D*) The frequency distributions of phages, lysogens, and total host cell populations with respect to biofilm cell packing for (*C*) the WT biofilm experiments and (*D*) the Δ*csgBA* biofilm experiments. (*E* and *F*) Quantitative comparisons of the mean cell packing and the SD in cell packing near lysogenized cells in comparison with the total host bacterial population over many runs of the experiments shown in (*A*−*D*). (*G* and *H*) Representative two-dimensional optical section images of experiments in which biofilms were treated as for (*A* and *B*) but were tracked for 120 h instead of 24 h. (*I*) Lysogen frequency over 120 h for the WT background and the Δ*csgBA* background. The shaded regions around the boxplots indicate the full range of data from all replicates. (*J*) Phage titer over 300 min when inoculated with no bacteria, naïve *E. coli*, lysogenized *E. coli* (phage resistant and phage-adsorbing), and Δ*lamB E. coli* (phage-resistant, but not phage-adsorbing. The dashed line indicates the limit of detection. (*K*) Bacterial CFU count for naïve host *E. coli* and lysogenized *E. coli*, as well as λ phage titer over 300 min when phages are introduced to a population of only naïve hosts in shaken liquid culture. For comparison, a separate control culture of phage-naïve *E. coli* with no phage added is shown as well. The dashed line indicates the limit of detection.

As biofilms increase in size under flow, cells may detach actively or passively from existing groups, reattach elsewhere in the chamber, and begin producing new cell clusters. As a result, after 48 h of incubation prior to adding phages, our biofilm chambers contained a variety of *E. coli* host cell groups of different size and packing architecture. In this culture condition, the largest and longest-established clusters of *E. coli* are each composed of a densely packed center, surrounded by cells with lower packing density (*SI Appendix*, Fig. S3). After phages were added to the flow devices, spatial heterogeneity in cell infection state (unexposed, lysed, lysogenized) arose within 24 h. Over this time scale, per expectation, the predominant mode of phage propagation was through lytic infection events in which phages produced a new burst of phage virions. Consistent with previous reports, we found that biofilm cell groups of relatively small size and low cell packing were lysed and removed from the system (12–14, 31). Larger, more densely packed clusters of *E. coli* were commonly found to have a peripheral region containing many λ phages, newly formed lysogens, and naïve host cells, as well as a highly packed central core of cells with few phages and no lysogenized hosts (Fig. 1 *A* and *C*). Previous work has shown that the *E. coli* matrix protein curli is essential for the cell–cell packing that can slow or halt phage diffusion (31). We reasoned that if curli matrix production is necessary for creating biofilm regions that are inaccessible to phages, a mutant strain of *E. coli* lacking the ability to produce curli should produce biofilms in which all cells are phage-accessible, and lysogens should arise relatively homogeneously throughout the system. We tested this possibility by repeating the experiments above using a strain of *E. coli* (denoted Δ*csgBA*) harboring deletions of *csgB* (encoding the curli baseplate) and *csgA* (encoding the curli amyloid monomer). This strain does not have a growth defect in liquid culture; however, its biofilms do not grow to the same height as those of WT, and their maximal cell packing is lower than that of the WT strain; these differences occur because Δ*csgBA* cells are not as well attached to each other and the underlying glass surface as WT curli-producing cells (69). When we repeated our experiment with biofilms produced by the Δ*csgBA* double mutant—consistent with our hypothesis and prior literature—phages and lysogens appeared to be homogeneously distributed among host cells (Fig. 1*B*).

To determine whether the naïve *E. coli* cells that survived did so due to de novo evolution of resistance to phage λ, we repeated the experiment above, but after introducing λ phages for 24 h, all of the cells within the microfluidic device were flushed out and plated for resistance to λ infection (*Methods Summary*). The frequency of λ resistance in this surviving *E. coli* population was approximately 10^-5^, which is not significantly different from the background frequency of resistant mutants in our overnight cultures used to inoculate the biofilm chambers at the start of the experiment (*SI Appendix*, Fig. S4). This result indicates that de novo genetic λ resistance did not contribute to the population dynamics in our experiments, and it is consistent with the fact that our biofilm cultures contain bacterial and phage population sizes and contact rates that—due to spatial constraint on bacterial and phage movement—are orders of magnitude lower than those found in shaken liquid batch culture (12, 31, 36). The results above instead suggest—consistent with our prior work exploring T7 phage propagation—that *E. coli* biofilm architecture can limit the diffusion of phages to a region along the periphery of cell groups and create a refuge against phage exposure in its core region (12, 31). The biofilm cell packing architecture that leads to phage protection only occurs if *E. coli* has sufficient time to grow prior to the introduction of phages; if phages are introduced from the beginning of biofilm growth, the spatial pattern of naïve cell distribution, cell packing, phage diffusion, and lysogen localization shown in Fig. 1 does not occur (*SI Appendix*, Fig. S5).

Our observations above suggested that for *E. coli* biofilms of sufficient size and architectural maturity, temperate phage propagation can occur along the peripheral regions of cell clusters, where cell packing is lowest. In general, cell packing increases with depth from the biofilms’ outer surface (*SI Appendix*, Fig. S6), but we will use cell packing as the key index for this study, as cell packing, rather than biofilm depth, is more directly mechanistically linked to phage localization. The lytic activity of the phages tends to reduce local cell packing even further, and it is within this region of reduced cell density that lysogens are found (*SI Appendix*, Fig. S7). To assess this visual intuition quantitatively, we used the BiofilmQ analysis framework to calculate the distribution of lysogenized cells with respect to local cell packing density and to compare their distribution with that of the total *E. coli* population (*Methods Summary*). We found that lysogens are indeed overrepresented in biofilm regions with lower packing density and are heavily skewed in this respect compared to the background total distribution of cell packing within large *E. coli* biofilm colonies (Fig. 1*C*). Interestingly, the frequency distribution of lysogens peaks at lower biofilm cell packing than the λ phages themselves, which indicates that lysogenization has yet to equilibrate with the spatial range of phage spread by 24 h. When we repeat this experiment with a Δ*csgBA* strain lacking curli production, the spatial distributions of phages and lysogens relative to local cell packing are indistinguishable from that of the entire host cell population (Fig. 1*D*). The spatial patterns of lysogen and phage localization for WT versus Δ*csgBA* host background were consistent across many runs of these experiments (Fig. 1 *E* and *F*), and we confirmed using liquid culture experiments that these results are not due to any differences between WT *E. coli* and Δ*csgBA E. coli* in terms of phage susceptibility or phage adsorption rate (*SI Appendix*, Fig. S8). Notably, we observed lysogens within Δ*csgBA* biofilms at a marginally higher average cell packing than we did for lysogens within WT biofilms. We speculate that this was due to the ability of WT biofilms to progressively halt phage diffusion as they approach regions of high cell packing; Δ*csgBA* biofilms, on the other hand, pose no or minimal obstacles to phage diffusion and allow them to enter their full volume and range of cell packing density.

The results thus far suggested to us that the spatial patterning of lysogenization was due to the cell packing architecture that *E. coli* produces, which blocks phage diffusion into the highly packed core regions of host biofilm colonies. Previous research documenting halted diffusion of T7 phages into *E. coli* biofilms showed that this protective phage blocking was a function of the packing structure generated by the secretion of curli matrix proteins (31). A difference we observe here is that *E. coli* biofilm cell groups become larger with rougher surface topography in our media conditions (M9 minimal with 0.5% maltose), as opposed to the flat mat architecture seen in the prior study of T7 diffusion in *E. coli* biofilms (which was done in 1% tryptone broth). To document curli production quantitatively in our culture conditions, we used a previously engineered strain of *E. coli* with a fluorescent transcriptional reporter inserted into the *csgBAC* operon (31). We found that *csgBAC* transcription increases with cell packing up until the very highest values of cell packing fraction that we measured (*SI Appendix*, Fig. S9*G*) (70, 71). We repeated these experiments with an *E. coli* strain that has a 6xHIS tag fused to the csgA monomer, which allows for immunostaining of the curli biofilm matrix. This epitope tag has been shown not to interfere with the functionality of the curli matrix (31). We observe a similar relationship between cell density and curli immunostaining as we did with the *csgBAC* transcriptional reporter (*SI Appendix*, Fig. S9*G*). In addition to documenting the production of curli matrix by *E. coli* in our culture conditions, we note that high *csgBAC* transcriptional activity at the tightly packed center of biofilms in our experiments suggests that cells on the interior remain physiologically active and are not avoiding phage infection due to nutrient starvation and quiescence. Previous work has also documented that, at the center of biofilms of the size order that we examine here, cells still have access to growth-limiting substrates and remain physiologically active (12, 72).

### Lysogen Abundance and Localization Equilibrate in Biofilms over Multiple Days.

Our experiments thus far suggest that by virtue of matrix secretion and cell packing architecture in established *E. coli* biofilms, the introduction of temperate phage λ leads to lytic proliferation in a finite peripheral region of sufficiently large, densely packed biofilm cell groups. An inevitable byproduct of this pattern is that lysogenized hosts tend to occur in the outer boundary regions of large biofilm clusters, where cell–cell packing is lower by default and is decreased further by lytic-cycle phage activity (*SI Appendix*, Fig. S7). We were next curious about the stability of these patterns through time.

We grew WT and *ΔcsgBA E. coli* biofilms for 48 h prior to introducing λ phages continuously for an additional 120 h, acquiring images of replicate biofilm chambers daily (Fig. 1 *G* and *H*). The frequency of lysogens within WT biofilms increased gradually before reaching a steady state at ~10% of the population after 96 h (Fig. 1*I*). Given the constant influx of phages into the system, and the fact that lysogens themselves grow and divide in the peripheral regions of biofilm with highest nutrient availability, it was not immediately clear why lysogenized cells would tend to equilibrate at 10% of the population rather than continuing to increase in relative abundance. The first and obvious factor limiting lysogenization—explored in Fig. 1—is that lysogen frequency can reach an upper limit within structurally mature biofilms due to phage diffusion constraints that limit what fraction of the original cell population is phage-exposed. As a result, uninfected cells within a cell group can replicate and keep pace with the growth of newly replicating λ lysogens around the cell group periphery. A second explanation, not mutually exclusive from the first, is that newly formed lysogens surrounding uninfected cells could create barriers against further phage propagation, as lysogenized cells can adsorb λ virions but are immune to superinfection (73, 74). An additional explanatory factor, also not mutually exclusive from the previous two, is that newly formed lysogens might be disproportionately predisposed to disperse from biofilms due to their spatial arrangement on the shear-exposed biofilm periphery. We assess the first two possibilities below and expand upon the third in subsequent sections.

To first assess the role of *E. coli* biofilm structure on lysogen proliferation over time, biofilms of Δ*csgBA E. coli* were grown for 48 h without phages, and then monitored for an additional 120 h under continuous λ exposure. As observed for the WT biofilms above, lysogens initially arise slowly and after 48 h comprise only a small fraction of the total population. By contrast, after 48 h of the phage exposure the relative abundance of lysogens within Δ*csgBA* host biofilms continues to increase sharply, departing more and more dramatically from the pattern seen for WT host biofilm structure. By 96 h after introduction of phages, lysogens constitute 80 to 100% of the total Δ*csgBA* population (Fig. 1*I*). This result makes it clear that matrix structure is a central factor controlling phage movement and replication, but it does not exclude the possibility that lysogens on the periphery of biofilms reduce further phage entry by adsorbing them.

To assess the adsorption of free λ phages to different host *E. coli*, we turned to well-mixed batch culture experiments in which λ phages were incubated with λ-susceptible naïve *E. coli*, λ-lysogenized *E. coli* (which can adsorb phages but do not support lytic propagation), Δ*lamB E. coli* (which lack the LamB maltose importer to which phage λ binds, and therefore do not adsorb phage λ), or a blank media control. We began these experiments with an initial phage:bacteria ratio of 1:10 (multiplicity of infection = 0.1) and tracked phage adsorption and amplification by quantifying free λ phage titer from the liquid media in each culture condition every 25 min for 5 h (*Methods Summary*). As expected, incubating phage λ in sterile media or with Δ*lamB E. coli* produced no change in phage titer throughout the experiment. When we incubated λ with a previously lysogenized *E. coli* population, we observed a rapid decline in liquid phage titer that fell below the limit of detection by 200 min; this follows expectation and recapitulates the classic superinfection immunity result (74–76). When we repeated this experiment with λ-susceptible *E. coli*, we observed an initial drop in phage titer as phages adsorbed to host cells. However, in this condition, the initial drop in phage titer is followed by a >100-fold increase corresponding to a burst of new phages from a round of lytic infection. As explored below, a rapid increase in new *E. coli* lysogens also occurs at this time (Fig. 1*J*). From this time point forward, the phage-host population dynamics become more complex, as the host *E. coli* population comprises naïve host cells and newly lysogenized hosts (both of which are actively growing), in addition to free λ phages. To better understand these dynamics, we repeated this experiment and quantified the changing abundances of lysogenized hosts and nonlysogenized naïve hosts by CFU count, in addition to monitoring free λ phage titer (*Methods Summary*). Lysogen abundance increases sharply straight from the start of the experiment, saturating until the release of new phages from the first round of lytic infection bursts from the initially naïve *E. coli* host cell population. With the release of this new λ phage burst, lysogen abundance again increases, due to growth of existing lysogens and new lysogenization of naïve cell hosts. Surprisingly, the nonlysogenized, uninfected naïve *E. coli* cells showed variance of less than 1 order of magnitude from their inoculated population size. Our interpretation of this result is that because the naïve cells are actively replicating and partially being converted to lysogens, which adsorb free phages, their populations remain relative stable despite successive rounds of phage release, adsorption, and lysogenization or lytic amplification (Fig. 1*K*).

Taken together, these results suggested that the steady state frequency of lysogens within naïve biofilms exposed to phages can be explained by phage diffusion limitation conferred by matrix-dependent cell packing structure, in addition to a possible contribution from phage shielding by lysogens that adsorb free λ virions. To assess this point in more detail, we sought to determine whether phage adsorption to lysogens could change phage mobility in the absence of dense packing structure that greatly reduces phage diffusion. We approached this problem by performing the following experiments in a Δ*csgBA* background lacking curli production. In one treatment, Δ*csgBA* cells (phage susceptible) were coinoculated 1:1 with a Δ*csgBA* λ lysogen (phage immune, adsorbs free phages). In the second treatment, Δ*csgBA* cells were coinoculated 1:1 with a Δ*csgBA*Δ*lamB* strain (phage immune, does not adsorb free phages due to lack of LamB receptor). By comparing the degree of phage localization to Δ*csgBA* cells in both of these conditions, where dense packing structure is absent, we can assess whether adsorption of phages to lysogens has an impact on phages’ ability to reach susceptible Δ*csgBA* cells. The biofilms were grown for 48 h and then exposed to phages for 24 h prior to imaging. We found that phage localization to Δ*csgBA* cells was significantly higher when they were cocultured with a nonphage adsorbing isogenic strain (Δ*csgBA*Δ*lamB*) than when they were cocultured with a phage-adsorbing isogenic strain (Δ*csgBA* lysogen) (*SI Appendix*, Fig. S10). These additional results indicate that the retention of phages at the surface of Δ*csgBA* lysogens can reduce phage exposure of a cohabiting susceptible strain. This result supports our hypothesis that adsorption of phages to lysogens’ cell surface can reduce phage mobility toward susceptible target cells. But, given that the effect of phage adsorption by lysogens can only be discerned when dense packing structure is removed, we conclude here that matrix-dependent impedance of phage diffusion is the dominant factor influencing the results noted in Fig. 1.

### Lysogenized Cells are More Likely to Disperse due to their Characteristic Spatial Arrangement.

So far, we have explored the process of phage propagation within individual biofilm patches as a function of the local cell packing architecture and the adsorption of phages by either naïve or lysogenized host cells as the biofilms grow toward steady state. We were also curious as to the flux of dispersed lysogens from biofilms’ exterior, particularly in light of the finding that lysogens tend to be created along the biofilm periphery in naïve biofilms exposed to phages. Given that lysogens are skewed in their spatial distribution toward peripheral regions of lower cell packing, and that ongoing lytic cycle activity by phage λ reduces local cell packing further in the regions where lysogens are located (*SI Appendix*, Fig. S7), we hypothesized that lysogens are disproportionately more likely to disperse from cell groups and recolonize elsewhere relative to their total frequency in biofilms.

**Fig. 2.**
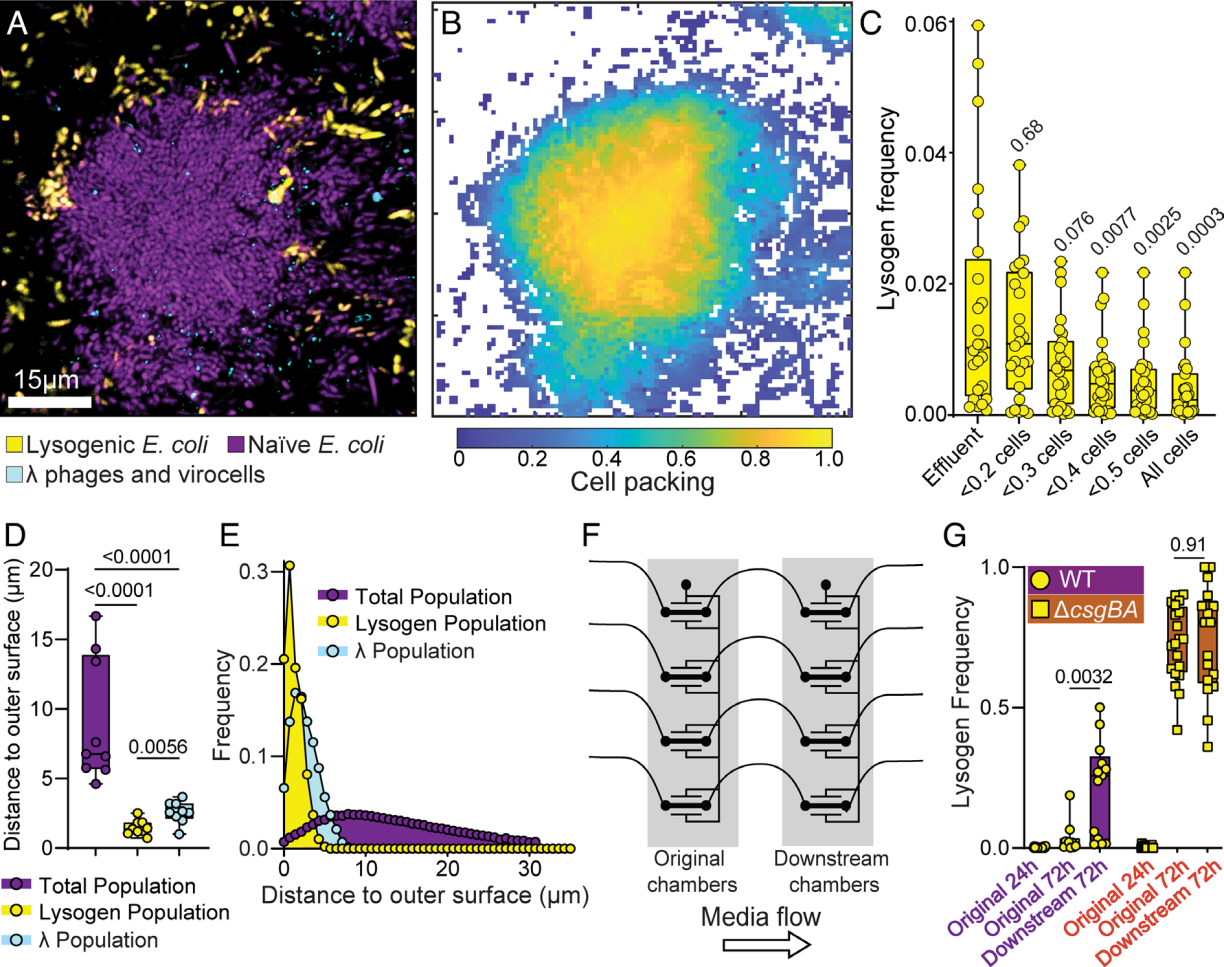
Lysogens are overrepresented in the liquid exiting chambers because they arise in peripheral biofilm regions with low packing density. (*A*) Representative two-dimensional optical section of a phage-naïve WT *E. coli* (purple) biofilms following exposure to phages (cyan). Lysogens (yellow) have been formed around the periphery of the biofilm cell group. This image is the bottom layer of a 3-D biofilm cluster—for full data analysis that follows, the entire 3-D biofilm’s volume is imaged and quantified. (*B*) A heatmap showing a two-dimensional projection of cell packing measurements for the full 3-dimensional z-stack of images capturing the group of cells shown in (*A*). Note the reduction in cell packing as one moves from the center to the edge of the colony. (*C*) A comparison of the frequency of lysogens in liquid effluent exiting biofilm chambers, relative to the frequency of lysogens in regions of different cell packing. Effluent lysogen frequency is closest to that in the regions of with average cell packing <0.2 on the periphery of biofilm clusters; this peripheral, loosely packed region of biofilm clusters makes the largest contribution to the dispersal pool. (*D*) Measurements of distance to outer cell cluster surface for the total prey cell population, lysogens, and phages. Phages and lysogens are restricted to the outermost layers of cells with low packing density. (*E*) Frequency distributions of distance to outer cell group surface for the total prey population, lysogens, and phages across all experimental replicates. (*F*) An illustration of the dispersal assay in which biofilms are first grown in the left hand (“original”) chamber, after which the effluent from this chamber is used to inoculate a second (“downstream”) chamber to simulate dispersal from one location to another. (*G*) The frequency of lysogens in the original upstream chamber and the new downstream chamber for dispersal experiments with the WT *E. coli* strain background and the isogenic Δ*csgBA* mutant that cannot produce curli matrix.

To test this idea experimentally, we incubated WT *E. coli* biofilms as in Fig. 1, inoculated them with phages for 24 h, and imaged the system to measure abundance of naïve and lysogenized cells (Fig. 2*A*). We then immediately collected effluent from these microfluidic devices and prepared the contents for imaging with no delay during which cell growth or changes in phage activity could occur. We calculated the relative abundance of lysogens in the liquid effluent and found that indeed the lysogen frequency in the exiting liquid phase is significantly higher than it is in the total biofilm population within the chambers (Fig. 2 *B* and *C* and *SI Appendix*, Fig. S11). Specifically, lysogens were 10-fold more likely to be dispersed than the average nonlysogenized naïve host cell in the system. On the other hand, if we isolate only the fraction of the population residing at a cell packing fraction of less than 0.2, then the lysogens’ frequency in this subpopulation is indistinguishable from its frequency in the effluent (Fig. 2*C* and *SI Appendix*, Fig. S11*D*). This observation reinforces the interpretation that the dispersing cells are derived primarily from the outermost, lower-density biofilm regions, which is where the lysogens are most abundant. Direct quantification confirms that phages and lysogens are indeed restricted to the outer layers of biofilms over many replicates of this experiment (Fig. 2 *D* and *E*). By conducting this same experiment after 72 h of phage exposure (as opposed to 24 h from the experiments above), we could show that the disparity between lysogen frequency in the effluent relative to that in the original biofilm is stable over multiple days following phage exposure (*SI Appendix*, Fig. S12*A*). This suggests that the pattern of biased dispersal of lysogens is a persistent feature of this experimental system. Note that we are not claiming here that phage exposure leads to greater dispersal of the overall biofilm, but rather that the spatial patterns of phage transport and lysogen production lead to a stable overrepresentation of lysogens relative to nonlysogens in the dispersal pool departing naïve biofilms exposed to phages.

As phage λ carries genes that can modify host outer membrane composition (77, 78), we checked whether λ lysogens are more likely to disperse due to a property conferred by the integrated phage genome itself. To do so we inoculated biofilm chambers with a 1:1 mixture of prelysogenized cells and naïve cells and tracked the biovolume of both strains within the chamber and within the effluent. We found that uninfected and lysogen cells compete neutrally when in biofilm culture together (*SI Appendix*, Fig. S12*B*); that is, net results of population growth, minus total cell deaths, minus cell dispersal events are the same for WT and lysogens when they are inoculated together at the start of the experiment. This experiment also showed that when phage-naïve WT *E. coli* and λ lysogens are inoculated 1:1 at the start of the experiment, there is no longer a difference in the fraction of lysogens in the biofilm chamber versus the fraction of lysogens in the liquid effluent from the chamber (*SI Appendix*, Fig. S12*B*). These data, together with all of the results we have shown thus far, imply that in the case when phages are added to an initially phage-naïve, nonlysogenized host biofilm, lysogens disperse more frequently than naïve cells not because of a change in biofilm dispersal physiology caused by prophages, but rather due to the spatial arrangement in which lysogens are generated.

The result that lysogens are especially disposed to disperse from naïve WT biofilms prompted us to consider larger spatial scale patterns of phage spread and biofilm distribution. We hypothesized that because lysogens are disproportionately more likely to depart from biofilms relative to nonlysogenized cells, the new biofilms reseeded by this dispersal pool downstream from the original population will be overrepresented for lysogens relative to biofilms from which the dispersed cells originated. We tested this possibility by growing biofilms as above for 48 h, adding phages continuously for 24 h, sampling effluent from these chambers, and inoculating the liquid effluent into fresh microfluidic chambers (Fig. 2*F*). This effluent contained mixtures of naïve cells, lysogenized cells, and free λ phages. After a 45 min attachment phase, we incubated these biofilms for 72 h without any additional phages being introduced to the flow devices. After 72 h of growth, the frequency of lysogens in the downstream chambers was significantly higher than what we observed in the original chambers (Fig. 2*G*). There was notably high variance in this result; we speculate that this is due to biological variation in the upstream biofilm population structure and the bottleneck recolonization events from the effluent samples, which deposited relatively small, noise-prone samples of cells on the glass in downstream biofilm chambers. Variation in the number of phages departing the original chambers and interacting with populations of bacteria on their way to the new chambers could have contributed to variation in downstream lysogen abundance as well. When we performed this same experiment with our Δ*csgBA* strain of *E. coli*, we saw no difference between the original chambers and downstream chambers at equivalent time points, again consistent with all of the analyses above (Fig. 2*G*).

Altogether, our data suggest that the natural spatial constraints of biofilm cell group architecture lead to a repeatable spatial pattern of lysogen generation when naïve biofilms are exposed to temperate phages. Lysogens tend to be concentrated in the periphery of established, densely packed biofilm clusters and equilibrate at ~10% of total population, because λ phages only rarely diffuse to areas past a characteristic cell packing threshold and are also neutralized by previously lysogenized cells. Concentrated on the periphery of biofilm clusters, where cell packing is natively low and decreases further due to lytic cycle phage activity (*SI Appendix*, Fig. S7), lysogens are inherently prone to disperse and become overrepresented in the pool of planktonic cells exiting the system. This leads to their overrepresentation in new biofilm populations seeded downstream of the populations from which they originated. The overall nature of temperate phage propagation by host lysis and lysogenization on the biofilm periphery inherently promotes the efficiency of spatial spread of lysogenized cells over the length scale of biofilm dispersal and recolonization.

### Successive Phage Propagation After Lytic Induction Is Blocked by High-Density Cell Packing in Biofilms.

**Fig. 3.**
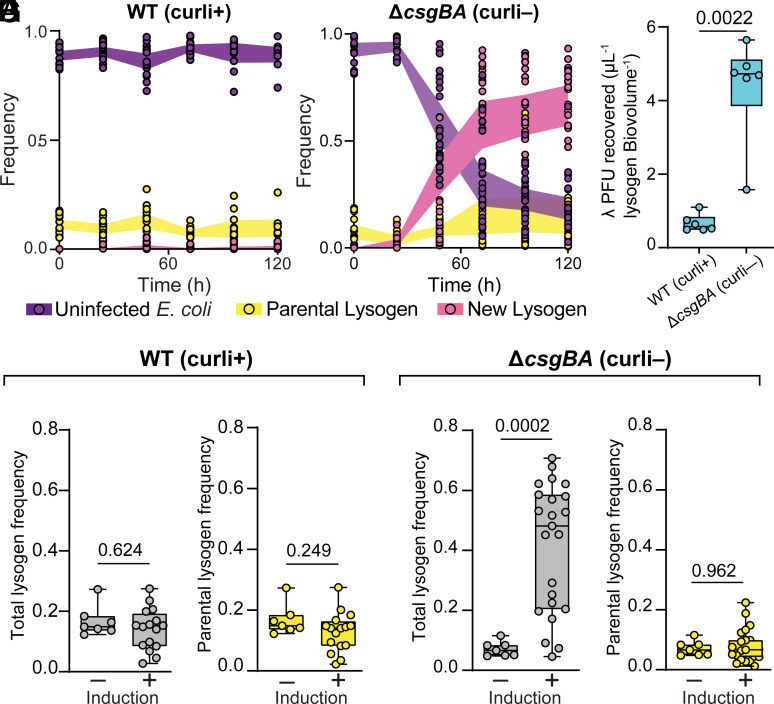
Phages released from lysogens within WT biofilms are not able to spread through the rest of the system; phages released from lysogens in a Δ*csgBA* background do spread through the rest of the population due to lack of high cell packing and diffusion impedance in Δ*csgBA* biofilms. Biofilms of either (*A*) the WT background or (*D*) the Δ*csgBA* background were inoculated with a 1:10 mixture of λ lysogens and naïve host *E. coli* and grown for 72 h. Lysogens were then induced to switch to the lytic cycle by incubating the biofilm chambers for at 42° C for 40 min. (*A*) The population dynamics tracked over 120 h for uninfected *E. coli* hosts, parental lysogens, and newly derived lysogens produced by phages released during the induction treatment at 48 h. (*B*) Total frequency of (parental + newly derived) lysogens, and the (*C*) frequency of only the newly formed lysogens at 120 h in WT biofilms with, or without, lytic induction of prophages. (*D*–*F*) Identical experiments as for (*A*–*C*), but in this instance using the Δ*csgBA* curli-null strain background for both the lysogen and the nonlysogen; without curli, phage diffusion is unimpeded, and a consequently large number of new lysogens are made that convert ~75% of the population into lysogens. (*G*) Quantification of phages exiting chambers of WT or Δ*csgBA* biofilms following heat induction to prompt the lytic switch among prophages. Phage diffusion is reduced or eliminated in WT biofilms, hence far fewer phages exit the chambers after induction; for the Δ*csgBA* background, whose biofilms do not block phage diffusion, a larger number of phages can be found exiting the chamber following lytic induction of prophages.

Our work thus far illustrates that while phage λ has limited ability to invade established biofilms of naïve cells, its pattern of lysogenizing hosts along the biofilm periphery leads to overrepresentation in the dispersal pool and frequently a large increase in relative abundance after colonizing new locations. We next asked: In biofilms initially colonized by a mixture of lysogens and naïve cells, does lytic induction of the lysogens allow phages to spread from within and infect hosts that they could not reach when introduced in the liquid phase? To assess this question, we first inoculated biofilms with a mixture of lysogens and naïve host *E. coli* at a ratio of 1:10 (Fig. 2*G*). Biofilms were grown for 72 h prior to applying heat to the chambers to induce lysogens to switch to the lytic cycle (*Methods Summary*). Note that only a minority of lysogens are induced to lytic phage production. In these experiments, naïve *E. coli* cells carried the constitutive mKate2 reporter, while the inoculated parental lysogens did not; this way, the parental lysogens (mKO2 fluorescent only) could be distinguished from new lysogens (mKate2 and mKO2 fluorescent) produced by phages released from lytic induction events. Following lytic induction, the population dynamics of inoculated lysogens, new lysogens, and remaining naïve cells were tracked daily for 120 h. When we performed this experiment in WT *E. coli* biofilms, we detected negligible production of new lysogens relative to a control case without heat induction (Fig. 3 *A*-*C*). This indicates that the limitation on phage diffusion observed in earlier sections still applies if phages are released from induced lysogens from within established biofilms.

We speculated that if we again relaxed the cell packing architecture of our experimental biofilms by using the Δ*csgBA* curli null background rather than WT, phages released from induced lysogens may be able to diffuse more freely and infect naïve cells with which the lysogens were inoculated. Supporting this idea, when we inoculated lysogens and naive cells of the Δ*csgBA* background and allowed them to grow before inducing the lysogens’ lytic switch, we observed a steep increase in new lysogen production, with lysogens ultimately reaching the majority of the population by 120 h (Fig. 3*D*). It is interesting to note that the expansion of the lysogen population in these experiments was driven predominantly by the production of new lysogens by released phages, rather than by the growth of the initially inoculated parental lysogens (Fig. 3 *E* and *F*). This suggests that time scale of phage transport through Δ*csgBA* biofilms—and the production of new lysogens by infection of exposed naïve cells—is substantially faster than the time scale of further growth by the parental lysogens over the 120 h we tracked the experiments following the lysogen lytic switch induction treatment.

The stark contrast between new lysogen production in WT versus Δ*csgBA* biofilms suggested to us that biofilm architecture coordinated through the expression of curli fibers can block successive rounds of infection and host lysogenization from within a biofilm, even when phages originate from lysogens coinoculated with naïve cells from the start of the experiment. In the absence of other mechanisms promoting phage mobility through biofilm cell groups, lytic induction of scattered lysogens in the biofilm population contributes little to production of new lysogens in a WT *E. coli* biofilm context. If the mechanism underlying our results is greater phage diffusion through the Δ*csgBA* background, we predict that when lysogens are induced to switch to lytic infection within biofilms of a Δ*csgBA* host, phages should be released into the surrounding liquid more readily than for WT biofilms. We tested this idea by performing the same lysogen induction experiment described above and sampling the liquid effluent of both WT and Δ*csgBA* biofilms for phage titer 3 h after lytic induction of the inoculated lysogens to allow for a full lytic induction cycle to occur at room temperature. We reasoned that if phages have higher mobility through Δ*csgBA* biofilms, they should escape more easily following induction, and we should thus recover higher titers of phage from these biofilms (normalized to lysogen abundance). As expected, we recovered ~10 fold higher phage titers from Δ*csgBA* biofilms than from WT *E. coli* biofilms (Fig. 3*G*) after inducing lysogens to switch to lytic phage production.

### Dissemination of Temperate Versus Obligately Lytic Phages within and Among Biofilms.

Thus far, we have seen that the temperate phage life history does not necessarily improve within-biofilm phage spread, but because of the spatial pattern of lysogen production in naïve biofilms exposed to phages, phage λ is predisposed to depart into the liquid phase and to reach much higher fractions of the population downstream during recolonization. These observations ultimately prompted us to ask: How do the net benefits and costs of the temperate phage life history for propagation in biofilms compare those of a purely lytic phage? To explore this question, we first performed experiments in which *E. coli* biofilms were grown for 48 h, and then inoculated these biofilms with either phage λ, λΔ*cI*, or T7 phages in separate experimental replicates. T7 is an obligately lytic phage that must kill its host in order to propagate. This strain of T7 contains a transcriptional fusion encoding *sfGFP*, which causes host cells to fluoresce green prior to lysis (31). We constructed the λΔ*cI* strain by replacing the cI locus encoding Repressor with a truncated nonfunctional copy; this phage retains the mTurquoise2 capsid decoration protein fusion, such that phage particles can still be tracked. cI is required for maintenance of lysogeny in λ lysogens. Without a functional copy of cI for Repressor synthesis, λΔ*cI* can only propagate by lytic infection (*SI Appendix*, Fig. S13).

We monitored phage propagation in biofilm chambers exposed to phages continuously for 7 h, then removed phages from media flowing into the chambers and returned sterile medium flow to them for 3 h to remove any phages that were not physically associated with the biofilm-dwelling host population. We then connected the effluent lines of these microfluidic devices to new downstream devices and allowed cells and phages to colonize the new chambers for 2 h; finally, new influent tubing was connected to the downstream chambers to introduce sterile media for an additional 96 h. We used infected/lysogenized cell biovolume as our metric for phage spread and propagation in our biofilm chambers, where cells can be determined to be infected if they are undergoing the lytic cell fate or if they are lysogenized. Assessing phage fitness using infected cell counts, including lysogens, has been established as the most robust metric for studying phage population dynamics in recent theoretical work (79–81).

**Fig. 4.**
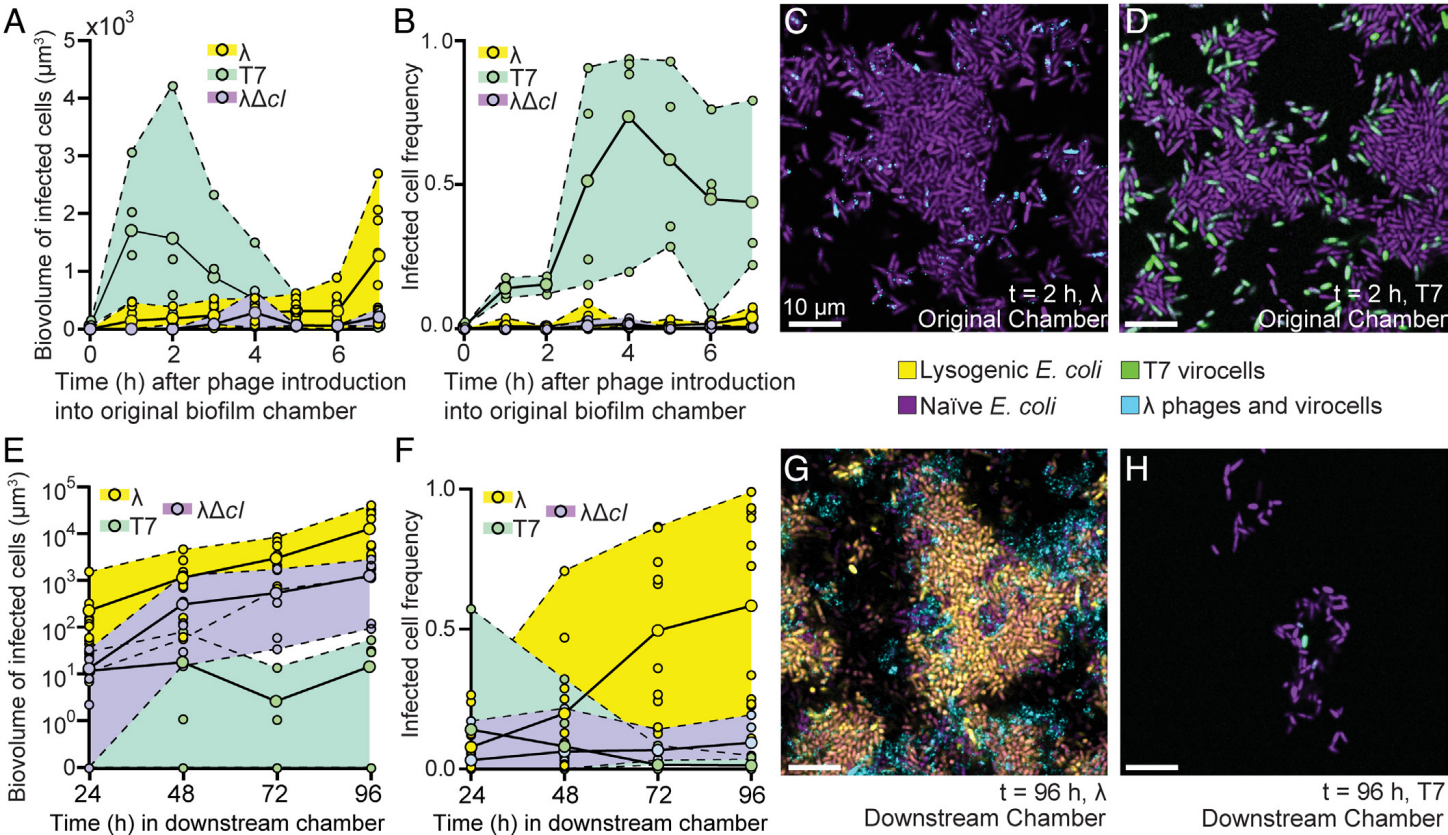
Phage T7 excels at rapidly exploiting available hosts in a large burst of lytic infection events. Phage λ however excels at increasing in relative abundance and self-sustaining within biofilm with period of dispersal and recolonization. Here, biofilms of WT *E. coli* were grown for 24 h before introduction of phage λ, T7, or a mutant of phage λ lacking Repressor; the latter phage was a control, as it is identical to λ but no longer lysogenizes hosts. After tracking local dynamics for 7 h, a dispersal cycle was implemented in which cells and phages exiting with the upstream chamber effluent were used to colonize new, sterile chambers (downstream chambers) (*A*) Biovolume of infected cells in original (upstream) microfluidic devices for 7 h of phage exposure to either T7, λ, or λΔ*cI* phages (n = 4 to 8). (*B*) Biovolume of infected cells in new (downstream) microfluidic devices over a 96 h time course (n = 4 to 8). (*C* and *D*) Representative two-dimensional optical sections of t = 2 h of exposure to λ or T7 phages, respectively. (*E*) Infected cell frequency as a proportion of the whole population for T7 or λ in original (upstream) microfluidic device (n = 4). (*F*) Infected cell frequency in new (downstream) microfluidic devices (n = 4 to 8). (*G* and *H*) Representative two-dimensional optical sections of t = 96 h in the new microfluidic devices.

In the first 7 h of exposure to phage T7, we see the highest accumulation of infected cells just 2 h after phages are inoculated into the system (Fig. 4 *A* and *D*). This is followed by a rapid decrease in infected cells, as the T7 phages kill off most of the cells in the chambers (Fig. 4 *A* and *D*). When we conduct this experiment with λ or λΔ*cI* phages, we observe qualitatively different population dynamics than for T7. Rather than an early peak followed by a subsequent decrease in infected cell biovolume, we see a slow increase in infected cell numbers, contributed to by both lytic and lysogenic infection events by phage λ, and only lytic infection events for λΔ*cI* (Fig. 4 *A* and *C*). If we instead measure the infected cell frequency as a function of the total population, we observe T7 phages reach their highest frequency within 4 h of inoculation in the original chamber, followed by a decrease as cells continue to lyse, exhausting the prey population. Infected cell frequency of λ and λΔ*cI* phages remain low during the 7 h in the original chamber (Fig. 4*B*).

To follow phage and host dynamics over larger spatial scales, we repeated the downstream chamber recolonization experiment in which effluent from original upstream chambers was used to inoculate new, sterile chambers. For phage T7, we occasionally found no cells—infected or uninfected—in downstream chambers (Fig. 4 *E* and *H*). More frequently, we found a small population of *E. coli* cells that had colonized the surface, with a small proportion of these cells actively infected with T7. As a result, the biovolume of infected cells for T7 is lower than for phage λ, and infected cells also make up a small fraction of the total population (Fig. 4*F*). In the downstream chambers exposed to λ phages, we observe an increase in infected cell biovolume, primarily driven by lysogenized cells continuing to divide and populate the new downstream biofilm (Fig. 4 *E* and *G*). The active phage particles in this system can also continue to lyse and lysogenize uninfected cells, further contributing to the increase in biovolume of infected cells and high infected cell frequency (Fig. 4*F*).

For downstream chambers of λΔ*cI*, we observed a lower infected cell frequency than for λ, but a higher biovolume of infected cells than for T7 (Fig. 4 *E* and *F*), which we attribute to T7 having a shorter lag time and higher burst size than phage λ lytic infection events. So, in the original chamber where phages were added continuously for 7 h, we observed T7 accumulating higher numbers of virocells than λ phages. However, in the downstream chambers, we see the opposite effect, where temperate phage λ accumulates 1,000-fold higher biovolume of infected cells relative to T7, and λ-infected cells dominate the population over extended time scales. This outcome indicates that virulent and temperate phage strategies are differentially suited to propagation on short time and small spatial scales (for quickly replicating virulent phages), versus propagation over longer time and larger spatial scales (for temperate phages).

## Conclusion

Live, high-resolution imaging of bacteria and phages dwelling in biofilms provides critical insight into the ecology of microbes on the spatial scale at which their behavior directly manifests. Here, we tracked the spatial propagation of temperate phage λ in sensitive *E. coli* biofilms, finding consistent patterns in the localization of phages and lysogens along the periphery of biofilm cell groups. Because of diffusion constraints imposed by biofilm cell packing architecture, phages can access only a modest fraction of susceptible *E. coli* in mature biofilms. Furthermore, even once lysogens are present in biofilms alongside susceptible hosts, lytic induction of the lysogens has little impact on promoting new infections, again due to the constraints on phage diffusion imposed by WT biofilm structure. On the other hand, the natural tendency for lysogens to be generated on the periphery of biofilms by phages in the surrounding liquid proved to be important for a different reason. Lysogens’ location on the outer boundaries of otherwise naïve biofilms, where biofilm cell packing is lower and decreased further by lytic phage infections, leads to preferential dispersal of lysogenized cells to new locations downstream of the original site of biofilm growth and phage exposure.

When phages encounter biofilms of uninfected cells, diffusion limitation and restriction of phage activity to the periphery of biofilm clusters is common to phage λ and to the purely lytic phages that we have tested previously. However, this is where the similarities between the two phage classes end, because the production of lysogens by λ leads to qualitatively different community dynamics than those observed under obligate lytic phage exposure. Because lysogens, by virtue of their location on the outside of biofilm clusters, are predisposed to dispersal from biofilms of origin, temperate phage infection, and lysogen production count greatly exceed purely lytic phage productivity over longer time scales and over larger spatial scales on which dispersed cells from one biofilm colonize new downstream populations. This is a unique benefit of the temperate phage life history strategy in comparison with that of obligately lytic phages, and the result emphasizes that examining phage infection dynamics in biofilms can yield fundamental insight into the adaptive benefits of different phage infection strategies that are impossible to infer from traditional shaken liquid cultures.

This study provides a direct view into the complex interactions of temperate phages and their host bacteria in a biofilm context. For simplicity, we explored a single species of bacteria and a single strain of phage in any given experiment; further studies can expand on these results by increasing ecological realism in terms of species richness within bacterial biofilm communities, as well as greater diversity among the phages introduced to these communities. While our lysogenized *E. coli* is indistinguishable from parental nonlysogens in terms of biofilm production, many temperate phages change host behavior by introducing novel functions to them via lysogeny, including alterations to metabolism and extracellular product secretion (23, 25). How the traits introduced to their hosts by prophages alter biofilm community dynamics remains a largely unexplored topic and a promising direction for future work. Detailed biophysical modeling of the relative rates of cell growth, cell death due to lysis, phage transport, lysogenic conversion, and cell dispersal will be helpful in defining the conditions under which our observations here are likely to generalize. Furthermore, assessing the generality of our findings with other examples of biofilm-forming bacterial species and their temperate phages will be important for the future, as will be assessing the consequences of our observations at larger community scales. Our results suggest, for example, that in environments where biofilm growth is commonplace, temperate phages should be especially abundant in comparison with lytic phages.

Last, we derived our inferences about the relative benefits and costs of the lytic and temperate phage strategies by growing each separately in different runs of our biofilm experiments. This is an important early step in differentiating how obligately lytic and temperate phages interact with their hosts in the biofilm context. However, in reality, there must be countless scenarios in which temperate and obligate lytic phages directly compete with each other in the same biofilm environments. The question of how temperate phages fare against obligate lytic phages in direct competition for hosts within and among biofilms is another crucial question for future research.

## Methods Summary

All bacterial strains were derived from *E. coli* AR3110. Biofilm growth was conducted using standard polydimethylsiloxane (PDMS) microfluidic devices, and all microscopy was performed using Zeiss 880 and 980 point-scanning confocal units. Data processing and analysis were performed using the BiofilQ analysis framework and MatLab. Prism was used for producing graphical figure panels and statistical analysis. Detailed methods are available in *SI Appendix*.

## Supplementary Material

Appendix 01 (PDF)

Dataset S01 (ZIP)

## Data Availability

All study data are included in the article and/or *SI Appendix*.
