# Supplementary material for: Spatial propagation of temperate phages within and among biofilms
Source: Proc Natl Acad Sci U S A. 2025 Feb 4;122(6):e2417058122. doi: 10.1073/pnas.2417058122 (PMC11831127; doi:10.1073/pnas.2417058122)
Supplement: Supplementary file 1 — Appendix 01 (PDF) [file pnas.2417058122.sapp.pdf]

Supplemental Information

Spatial propagation of temperate phages within and among biofilms

James B. Winans[1,2], Lanying Zeng[3], Carey D. Nadell[1,2*]

Department of Biological Sciences, Dartmouth, Hanover, NH

2. Department of Microbiology and Immunology, Geisel School of Medicine at Dartmouth, Hanover ,NH

3. Department of Biochemistry and Biophysics, Center for Phage Technology, Texas A&M University, College Station, TX

* Author for correspondence:

Carey Nadell (ORCID 0000-0003-1751-4895)

carey.d.nadell@dartmouth.edu

78 College St., Rm. 326

Dartmouth, Dept. of Biological Sciences

Hanover, NH 03755

Supplemental Methods

Strains

E. coli strains used in this project were all AR3110 derivatives. AR3110 was derived from the K-12 strain W3110, which does not produce cellulose because of a polar stop codon mutation in *bcsQ*. This stop codon mutation was corrected in AR3110 to yield a strain that produces cellulose, thus restoring this *E. coli* strain to "wild-type" biofilm formation secreting cellulose and curli protein (1). Like other K-12 derivatives, AR3110 is susceptible to both T7 phages and λ phages. Strains of AR3110 were engineered via lambda red recombination or through SacB counterselection allelic exchange. Briefly, primers encoding regions of homology to the flanking regions of the gene of interest were used to amplify the Kan^R resistance cassette flanked by FRT sites. For creating fluorescent strains, these resistance cassettes were fused to fluorescent protein constructs via SOE PCR. These PCR products were used to knock out genes of interest and selected for with the resistance cassette. FRT sites were used to flip-out the resistance cassette if desired. SacB allelic exchange was performed by constructing plasmids containing regions of homology to flanking regions and gene insertion, SacB, and a selective resistance marker. Plasmids were transformed into *E. coli* through electroporation and screened for kanamycin resistance, then counter-selected on sucrose plates to remove the antibiotic marker and native allele. Recombinant T7 phages were produced previously using T7select415-1 phage display. Recombinant λ phages were produced by infecting λD*am* cI_{857} *bor::KanR* phages on LE392 (permissive host) with the pBR322-λD-mTurquoise2/mNeongreen-E plasmid to allow for recombination, and further selection for fluorescent plaques. λΔ*cI* was created via lambda-red recombination that introduced a truncated nonfunctional copy of *cI* followed by screening for clear plaques (2). Newly generated strains for this paper will be available upon request.

Microfluidic flow device fabrication

Microfluidic devices were produced by casting poly-dimethysiloxane (PDMS; Dow Chemical Company, SYLGARD 184, cat. # 04019862) onto premade device molds (a schematic for this can be found in **Supplementary Fig. 1)**. The resulting PDMS blocks were cut out of the molds, hole-punched for inlet and outlet channels, and then bonded to #1.5 glass coverslips using plasma cleaning preparation of the PDMS and glass coverslips (Azer Scientific, cat. # 1152260). Between the inlet and outlet port areas, the internal space of the chambers in which biofilms were incubated measured 5,000 µm × 500 µm × 70 µm (LxWxH). Segments of inlet tubing (Cole Parmer PTFE #30, cat. # 06417–11) attached to 27Gx1/2 needles (BD Precision, cat. # 305109) on 1 milliliter syringes (Brandzig, cat. #CMD2583) were plumbed into chamber inlets, and the syringes were pushed by Harvard Apparatus Pico Plus Elite syringe pumps (Harvard Apparatus, cat. # 70–4506). Tubing from chamber outlet channels was fed to effluent waste collection.

Biofilm culture conditions

Non-lysogenized *E. coli* AR3110 were grown in liquid culture with shaking overnight at 37° C in LB broth. Lysogenized strains of *E. coli*, due to the temperature sensitivity of the Repressor protein in this strain, were grown in liquid culture with shaking overnight at 30° C. Cultures were removed from the incubators and allowed to cool to room temperature the following day before proceeding. Culture densities were measured by spectrophotometer, and cultures were OD-normalized to 1.0. If more than one strain was being introduced to the chambers, a combination of the respective overnight cultures was made in order to introduce a homogeneous mixture into the chambers for surface attachment. After a 45-min incubation period without flow to allow for surface attachment, M9 minimal media with 0.5% maltose continuously flowed into the device at a rate of 0.1 µL/min. For the immunostaining of curli, our AR3110 strain harboring a translational 6xHis tag fused to csgA, which encodes the monomer for curli production, was stained with Anti-6X His Epitope Tag (Rabbit) antibody conjugated to Dylight 405 (Rockland Immunochemicals, cat. # 600-446-382) added to the media at a concentration of 0.1 µg/mL continuously for the entirety of the experiment. Prior work has shown that addition of the 6xHis tag to CsgA does not interrupt its function by any measures tested (3,4). All biofilm experiments throughout the paper were carried out at room temperature (liquid culture phage-host interaction experiments in Figure 1J-K were also carried out at room temperature). Room temperature was chosen for three main reasons:

most importantly, *E. coli* upregulates biofilm matrix production at temperatures lower than 30° C. Second, we aimed here to represent a simplified abiotic environment *E. coli* might occupy, rather than the human gut. Third, microfluidic culture devices tend to be far more stable at room temperature, minimizing the occurrence of bubbles that would otherwise destroy the biofilm structure in the flow devices.

Note that *E. coli* grows in an open 3-dimensional space in our experiments, and biofilms were imaged to capture their entire volume (see 'Microscopy and image analysis' below). Many representative images in the main text are single 2-dimensional optical sections through the 3-dimensional biofilm image stacks; 2-dimensional images were usually selected for clarity, and to show the biofilm internal activity in addition to the activity on its outer surface area. Note however that all biofilms and image data sets refer to the full 3-dimensional system throughout the paper.

Phage propagation and introduction to biofilms

λ phages were produced by growing lysogenic *E. coli* to an OD_{600} = 0.2 at 30° C in M9 minimal media with 0.5% maltose, then heat shocked at 42° C for 20 min, and then incubated at 37° C until visible lysis occurred. T7 phages were produced by growing sensitive E. coli to OD_{600} = 0.4 at 37° C in M9 minimal media with 0.5% maltose, before adding an aliquot of T7 phage and incubating until the bacterial cultures were cleared. These protocol details were designed to extract as many phages from the host cell culture as possible for use in subsequent experiments. Phages were quantified using standard plaquing techniques and back-diluted to 10^4 PFU/μL in M9 with 0.5% maltose. To visualize T7 phage infection, we used a previously constructed T7 strain that induces sfGFP production by the host prior to lysis (4). For experiments with short phage exposure, phages were continuously introduced for 24 h. For experiments with extended phage exposure, phages were continuously added for 120 h. For the nascent biofilm phage exposures investigating spatial patterning in the absence of established biofilms, phages were introduced into the chamber immediately following the initial bacterial attachment step at the start of biofilm growth.

Biofilm dispersal and detection of *de novo* λ-resistance mutants from biofilm culture

E. coli biofilms grown for 48 h and then treated with λΔ*cI* phages for 24 h were dispersed from the biofilm by removing the tubing from the microfluidic device and vigorously pipetting 100 μL of M9 media and air bubbles back and forth between the inlet and outlet ports. This was done to ensure maximal removal of all cells in the chamber in order to capture accurate measurements of total *E. coli* and any de novo λ-resistant *E. coli*. To determine cell viability and phage sensitivity, an aliquot of this 100 μL volume containing dispersed biofilm cells was serially diluted and plated on LB plates for total *E. coli* counts and, in parallel, an aliquot was incubated with 10^5 PFU μL^{-1} λΔ*cI* phages for 30 min, and plated on LB plates saturated with λΔ*cI* phages to determine *de novo* λ-resistant counts.

Phage adsorption assay

Bacterial cultures of uninfected *E. coli*, lysogenic *E. coli*, and *E. coli ΔlamB* were grown until they reached exponential growth phase and back-diluted to an OD_{600} = 0.2 in λ medium. λ phages were added to a final concentration of 5×10^3 phages per μL. Cultures were incubated at 30˚C on an orbital shaking platform, and 200 μL aliquots were taken every 25 min, passed through a 0.2-μm filter, and stored on ice until the end of the experiment. The filtration step served to exclude any bacterial cells, and any phages that were attached to them, allowing us to measure free phages remaining in the liquid medium. Flowthrough samples were then serially diluted and plated for PFUs.

Phage adsorption and *E. coli* population dynamics assay

Sensitive *E. coli* was grown and back-diluted to an OD_{600} = 0.2 in λ medium. λ medium was used in these experiments (along with the adsorption assay described above) due to the experimental requirement for high-time resolution imaging over the full course of bacterial and phage population dynamics. Using λ medium allowed these experiments to proceed within the course of one day. λ phages were added to a final concentration of 5×10^3 phages per μL. Cultures were again incubated at 30° C on an orbital shaking platform, and 200 μL aliquots were taken every 25 min, passed through a 0.2-μm filter, and stored on ice until the end of the experiment.

Another 20μL aliquot was taken and directly added to a dilution series in a 96-well plate. To avoid *E. coli* amplification after sampling, these samples were immediately serially diluted and plated for CFUs. After all of the samples were collected, flowthrough phage samples were serially diluted for PFUs. To determine phage adsorption onto lysogenic cells within biofilms in the absence of curli production, biofilms of uninfected cells and lysogenic cells in a Δ*csgBA* genetic background were grown at a 1:1 ratio for 48 h prior to phage addition for 2 h. Biofilms were then imaged for phage localization around the two strains. This experiment was also performed with uninfected cells and Δ*lamB* cells, which do not have the λ phage receptor.

Biofilm effluent and recolonization assay

Biofilms were incubated for 48 h without phage exposure, treated with phage for 24 h, and then imaged. We then shortened the effluent tubing to allow for effective collection, and increased flow to 1 μL/min to collect 10uL of media. A portion of this sample was imaged under an agar pad to determine lysogen abundance. Another portion of this sample was used to inoculate new microfluidic devices, incubated without the addition of exogenous phages. These new microfluidic devices were tracked through time.

Lysogen induction assay

Naive, phage-sensitive *E. coli* and lysogenic *E. coli* were inoculated into biofilm chambers at a ratio of 10:1 for 72 h. Our λ phage carries the heat-sensitive *cI*857 allele; to induce lysogens to convert to the lytic cycle, microfluidic devices were placed into a 42° C incubator for 40min. This was sufficient to induce some, but not all, of the lysogenized cells into lytic phage production. Biofilms were then tracked for 120 h to measure the population dynamics of sensitive cells, parental lysogens, and new lysogens. New lysogen lineages – i.e., those that were created within the biofilm by phages released by the initially inoculated lysogens – can be differentiated from parental lysogen lineages, as new lysogens produce two different fluorescent proteins (mKate2 and mKO2) while parental lysogens only produce one fluorescent protein (mKO2). This, however, does not allow for differentiation between novel lysogenic infections and asexual bacterial replication or newly lysogenized hosts. In order to identify how biofilm structure is important for phage propagation, this experiment was carried out in a WT *E. coli* biofilm background, as well as a *ΔcsgBA* genetic background that cannot produce curli matrix proteins.

Phage mobility assay

In order to determine phage mobility on a global scale, Biofilms of lysogenic *E. coli* and Δ*lamB E. coli* in a WT background and *ΔcsgBA* background were grown at a ratio of 10:1 for 72h, quantified for lysogen biovolume, and lysogens were induced at 42° C for 40min. Effluent was collected from the microfluidic devices and PFUs were quantified and normalized to lysogen abundance in the biofilm chambers.

Microscopy and image analysis

All imaging was performed using a Zeiss 980 line-scanning confocal microscope, using a 40x/1.2 N.A. water objective or a 10x/.4 N.A. water objective. The 6xHis Tag Antibody Dylight 405 that was used to stain 6xHis-tagged curli polymers was excited with a 405 laser line. The mTurquoise2 protein that λ and λΔ*cI* phage capsid produces was excited with the 458 laser line. The sfGFP protein produced by the T7 infection reporter construct was excited with a 488 laser line. The mKO2 protein that lysogenic *E. coli* expresses constitutively was excited with a 543 laser line. The mKate2 protein that WT *E. coli* and Δ*csgBA E. coli* express constitutively was excited with a 594 laser line (in separate experiments). If one image was not sufficient to capture the variation seen within a given microfluidic device, multiple independent locations were chosen within each biofilm chamber and averaged to give 1 biological replicate measurement for a given chamber in the case of whole-biofilm measurements. Prior to export, images were processed by constrained iterative deconvolution in ZEN blue. Raw image data was then exported from Zen to the Biofilm Q image processing framework. Constitutive reporters for marking different strains, phages, and lysogens, were binarized using Otsu or Robust Background thresholding with a manual sensitivity parameter. For all analyses, a 3-dimensional grid was used to partition the segmented biovolumes into pseudo-cell cubes that were 0.72 μm on a side. Cell packing measurements merged the biovolume of all bacteria within a sample and calculated the biovolume fraction within 6 μm of each segmented bacterial volume within each grid cube. To measure *csgBAC* transcription for Supplemental Fig. 10, we quantified the *csgBAC* fluorescent reporter fluorescence inside each segmented volume of *E. coli*. To measure curli protein

immunostaining, we measured curli immunofluorescence in a 0.5 µm extending away from the outer surface of every segmented volume of *E. coli.*

Replication, quantification, and statistics

Replication is reported for each experiment individually in the legends of all of the figures. The reported sample size for each figure panel refers to biological replicates. One biological replicate was defined as the averaged outcome for measurements across a single microfluidic flow chamber inoculated from independent overnight culture preparations. Biological replicates for the core experiments in the study were performed across 3 weeks with independent microfluidic chambers. Technical replicates were separate z-stacks captured at randomized locations throughout a given flow chamber; measurements from these technical replicates were averaged to calculate the value for the biological replicate corresponding to that flow chamber. Mann–Whitney U tests with the Bonferroni correction were used for pairwise comparisons. We chose nonparametric comparison tests because they are relatively conservative and because the assumptions required for parametric tests could not consistently be assessed for our data. Boxplots denote the median, interquartile range, and range limit values; individual data points are shown where possible as well.

Table S1. Reagents, products, and software used in this study

**Strain**	**Relevant markers/Genotype**	**Source**
Bacteria		
CNE336	AR3110, with csgBAC-mKate2 transcriptional fusion, ptac-mKO-κ and KanR at attB site	(4)
CNE859	AR3110, mTurquoise2/mKO2 λ, 6x-*His* csgA	This study
CNE863	AR3110, *Lac::mKatex2*, *6x-His csgA*, AmpR-gpD plasmid	This study
CNE866	AR3110, mTurquoise2/mKO2 λ, 6x-*His* csgA, AmpR-gpD	This study
CNE869	AR3110, *Lac::mKatex2*, *ΔcsgBA*::KanR, AmpR-gpD plasmid	This study
CNE881	AR3110, Δl*amB::KanR, mKate2*	This study
CNE905	AR3110, sfGFP, Δ*lamB*	This study
CNE907	AR3110, Δl*amB, sfGFP, ΔcsgBA*::KanR	This study
CNE908	AR3110, Δ*csgBA::KanR*, mKate2, mTurquoise2/mKO2 λ, AmpR-gpD plasmid	This study
CNE909	AR3110, mTurquoise2/mKO2 λ, AmpR-gpD plasmid, pSIM5-tetR	This study
CNE913	AR3110, Δ*csgBA::KanR,* mTurquoise2/mKO2 λ, 6x-*His* csgA, AmpR-gpD	This study
Phages		
CNX11	T7 with sfGFP under control of phi 10 promoter	(4)
λLZ1367	*λD-mTurquoise2* cI_{857}*-mKO2 bor::CmR*	(5)
CNX20	*λD-mTurquoise2,* truncated $cI_{1\text{-}17::223\text{-}239}$ *, bor::CmR*	This study
**Chemicals and reagents**	**Source**	**Product number**

Kanamycin	Millipore-Sigma	cat.#60615
MEM Vitamin Solutions	Millipore-Sigma	cat. #M6895
Alexa Fluor™ 633 NHS Ester	ThermoFisher Scientific	cat. # A20005
Anti-6X His Epitope Tag (Rabbit) antibody conjugated to Dylight 405	Rockland Immunochemicals	cat. # 600-446-382
Poly-dimethysiloxane (PDMS)	Dow Chemical Company; SYLGARD 184	cat. # 04019862
#1.5 glass coverslips	Azer Scientific	cat. # 1152260
Inlet tubing	Cole Parmer	cat. # 06417-11
27Gx1/2 needles	BD Precision	cat. # 30510
1mL syringes	Brandzig	cat. #CMD2583
Harvard Apparatus Pico Plus Elite syringe pumps	Harvard Apparatus	cat. # 70-4506
**Software and Algorithms**	**Source**	**Version**
Zen Black	Zeiss	v14.0.0.0
Zen Blue	Zeiss	v3.4.91.00000
MATLAB	MathWorks	vR2021a
Paraview	Kitware	v5.9.1
Prism	GraphPad	v9.4.1
BiofilmQ	(61)	v0.2.2

Supplemental Figures

A

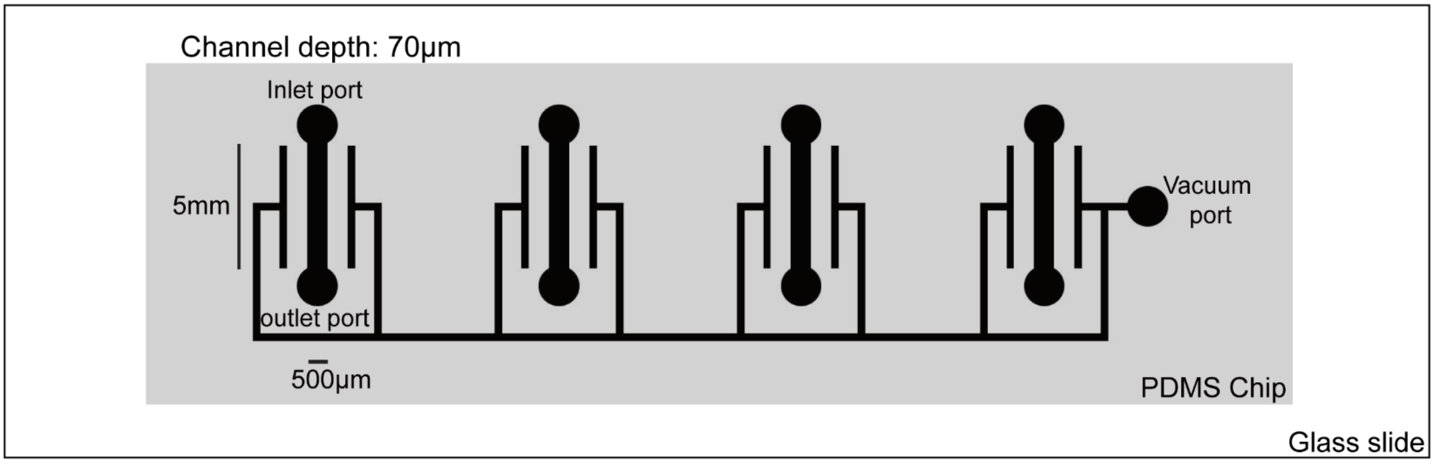

SI Figure 1. Diagram of our microfluidic devices. (A) This example contains 4 parallel chambers, each with an inlet and an outlet port for connection to fluid inlet/outlet tubing. Technical replicate image stacks were taken from within the straight rectangular section between the rounded inlet and outlet ports of a chamber. The thinner, continuous channel surrounding the 4 separated chambers was connected to a wall vacuum line to apply negative pressure; this method discourages the introduction of air bubbles into the liquid-filled portions of the flow chambers.

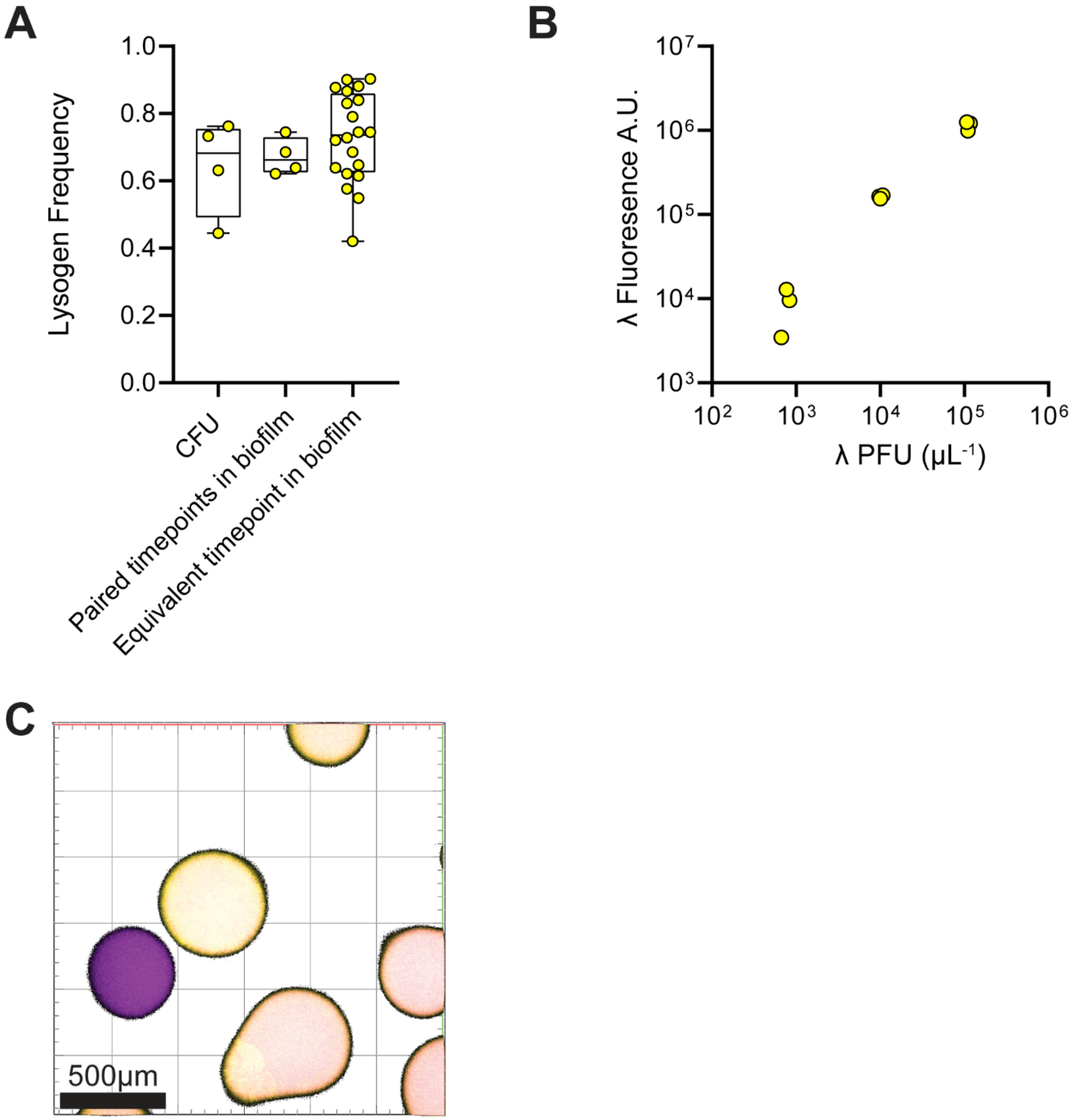

SI Figure 2. Control experiments documenting that our microscopy-based experiment techniques can quantify labeled phage abundance accurately. (A) Lysogen frequency measured by CFU selective plating after mass dispersal of biofilms from the microfluidic devices, compared to the lysogen frequency observed in the microfluidic devices measured by image quantification with BiofilmQ (n = 4–20). (B) Linear relationship between λ phage fluorescence and λ PFU (n=9) (C) Lysogenic *E. coli* colonies (yellow) on LB agar plates can be distinguished from naïve *E. coli* colonies (purple) by fluorescence. These measures all indicate that our imaging-based approach gives excellent fidelity to numerical counts of phages by traditional plating methods.

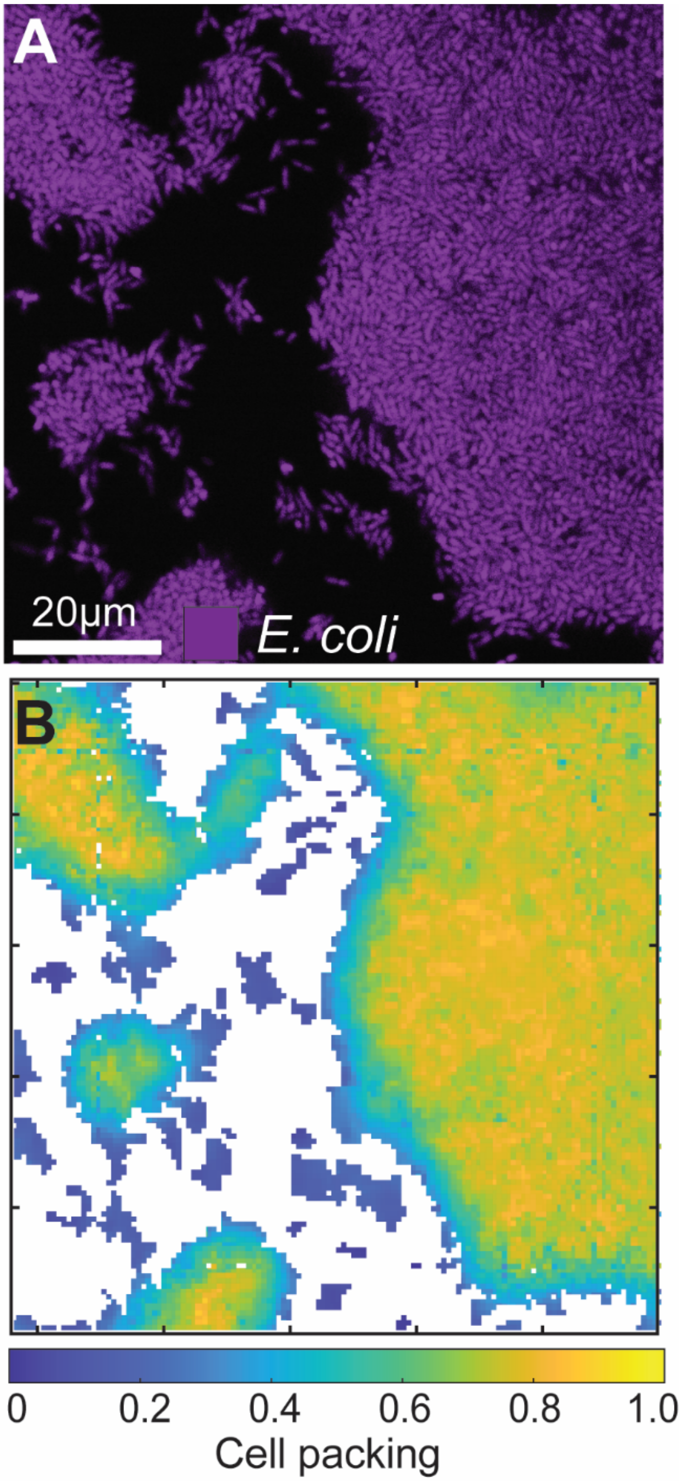

SI Figure 3. An illustration of typical *E. coli* AR3110 biofilm structure and cell packing in the absence of phage exposure. (A) Representative image of an *E. coli* biofilm prior to the addition of λ phages. (B) Heatmap of localized cell packing of (A), showing the spatial pattern most seen in *E. coli* biofilm cell packing after 48 h of growth. The heatmap is a 2-D projection of the analysis for the full 3-D z-stack of images captured.

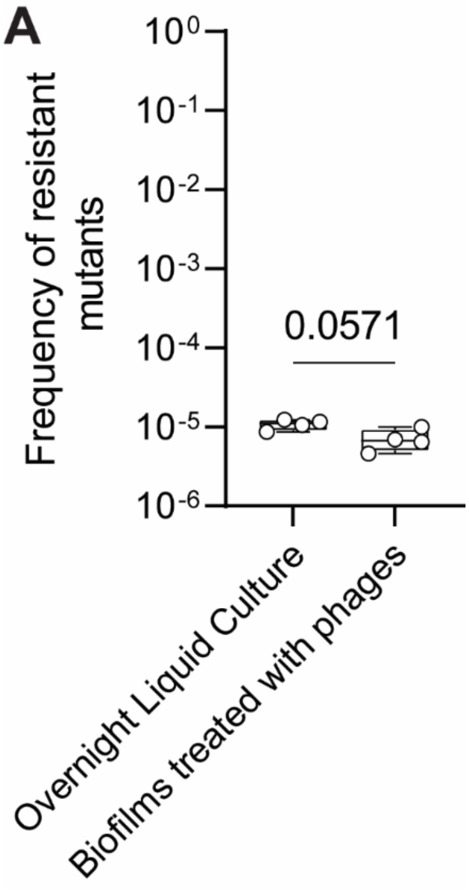

SI Figure 4. A test to determine if genetic resistance to phage λ spontaneously evolved during our experiments. *E. coli* was grown for 16 h in LB media at 37Cº overnight. To calculate the standing frequency of λ phage resistance, we plated one serial dilution series of this overnight culture onto plain LB agar; another aliquot was taken and mixed with10^5 PFU μL^{-1} λΔ*cI* phages for 30 minutes in liquid and then plated in serial dilution onto LB plates saturated with λΔ*cI* phages (i.e., phages that only lyse hosts). Adding phages to the liquid phase and to the plates ensures complete or nearly complete phage exposure of the sample bacterial population. The CFU counts on phage plates relative to CFU counts on plain LB plates gives the frequency of λ resistant mutants arising by spontaneous mutation in the overnight culture (10^{-5}). If host phage resistance frequency is higher than this value at the end of biofilm experiments with λΔ*cI* phage exposure, this would indicate that positive selection for genetic phage resistance is occurring in the biofilm experiments. To test this, we grew WT *E. coli* biofilms for 48 h, treated them with continuous λΔ*cI* phage, and then flushed the chambers to remove all cells. We then performed the same dual plating procedure with these samples as we did for the overnight cultures used to inoculate the chambers initially. That is, we plated one serial dilution on plain LB, and another aliquot was exposed to phages in liquid for 30 minutes prior to being plated on phage-saturated LB plate. The frequency of resistant mutants in the biofilm chambers following lytic λ exposure was actually slightly lower, but not significantly different from the initial frequency of resistant mutants from the overnight culture used to inoculate the chambers. This illustrates definitively that there was no detectable positive selection for λ phage resistance during our experiments.

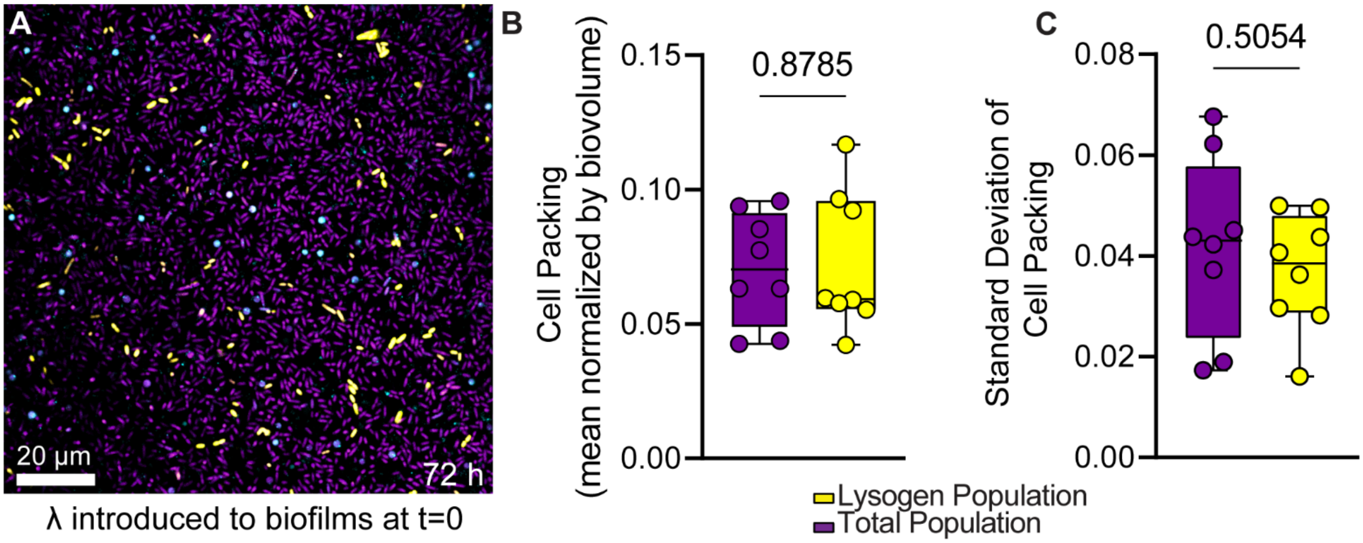

SI Figure 5. The distinctive patterns of phage and lysogen localization described in the main text are contingent on WT *E. coli* establishing its biofilm structure before introducing phages into the system. (A) λ phages introduced to *E. coli* biofilms at t=0 (i.e. at the same time as bacterial inoculation) do not exhibit the characteristic pattern of phage spread and lysogenization as observed in established WT *E. coli* biofilms (see Figure 1, Main Text). (B) A pairwise comparison of cell packing of the total population versus only the lysogenic population, which are not significantly different if phages are added to the flow chambers immediately after the start of biofilm growth (n=8). (C) The standard deviation of cell packing for the total population versus the same measure for only lysogenic population; these also do not differ (n=8), in contrast with the main results in Figure 1 of the main text, where the host was allowed to grow for 48 h prior to phage introduction.

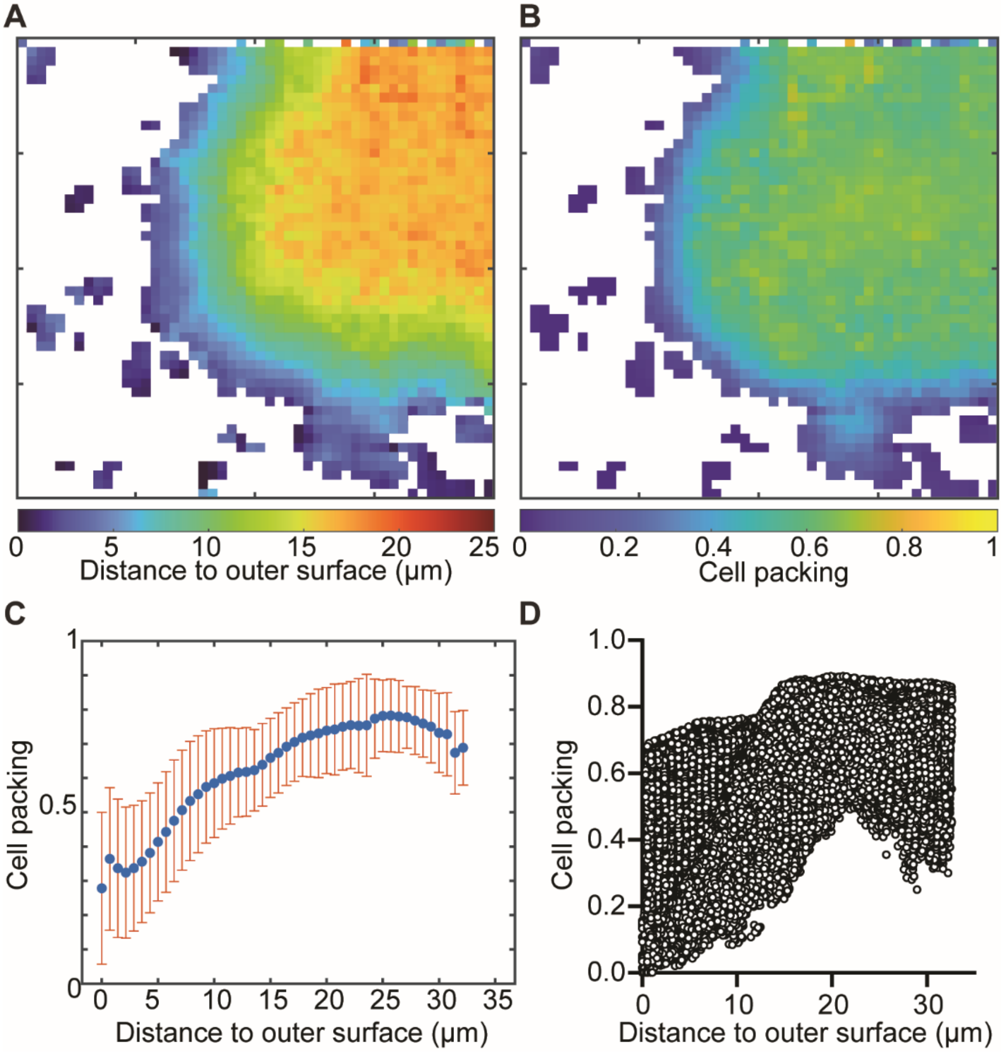

SI Figure 6. Cell position relative to the center of micro-colonies has a consistent correspondence with their nearby cell packing density. (A) Representative heatmap of distance to outer surface as a function of location in a typical biofilm cluster. (B) Representative heatmap of cell packing for the same image data as in (A). (C) A plot of cell packing as a function of distance to outer surface (n=9), illustrating the visual intuition that cell packing systematically declines with distance from the colony center. (D) A plot of cell packing as a function of distance to outer surface for each segmented volume of *E. coli* in replicate image data sets. Note that segmented bacterial volumes are partitioned into a 3-dimensional grid to delineate cell-sized objects (which in this case, are the volumes within each cubic node in the 3-dimensional grid) and to create a reference frame for spatial analysis (n=9, total segmented volumes of *E. coli* represented from the 9 image stacks = 234,695). We use the cell packing measurement as the key index of the paper, as this measurement is more generally applicable across species that don't have round biofilm colonies, and because it is more concretely linked to the mechanism underlying phage diffusion impedance.

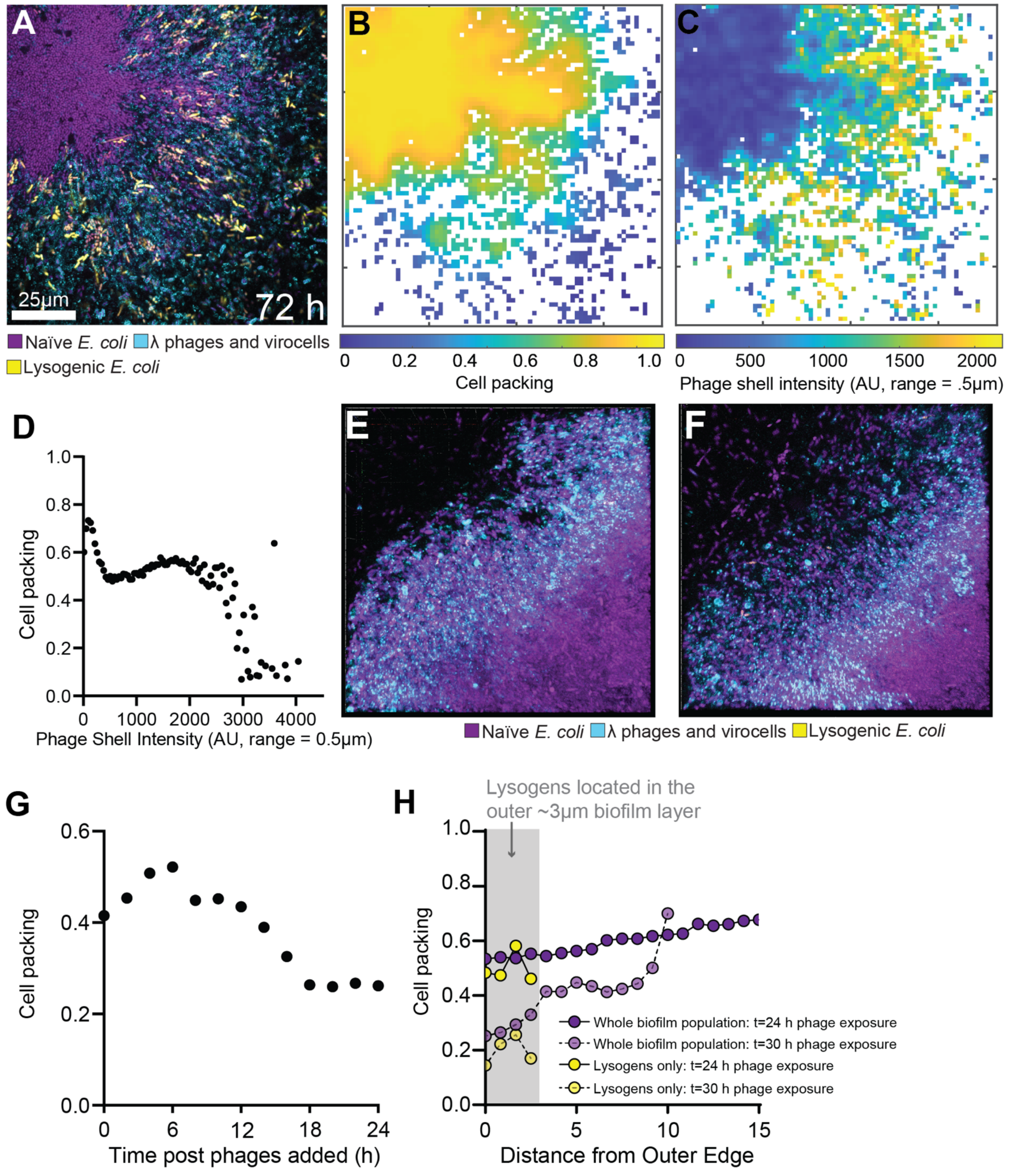

SI Figure 7. Quantitative documentation that phage λ lytic cycle activity reduces cell packing in the areas immediately surrounding newly created λ lysogens in WT biofilms. (A) Representative image of an *E. coli* biofilm grown for 48 h and then exposed to phages for 72 h. Naïve *E. coli* hosts are shown in purple, phages in cyan, and lysogens in yellow. (B) Corresponding spatial heatmap of cell packing for the biofilms shown in panel (A). (C) Spatial heatmap of phage fluorescence intensity corresponding to phage localization. Note the resitrction of their activity to the biofilm periphery, which is also where lysogens arte created. (D) Local cell packing as a function of local phage fluorescence intensity; this plot

indicates that higher local phage presence corresponds with lower cell packing. (E) The first time point (following 48 h biofilm growth and then 24 h phage exposure) of a high resolution timelapse of WT *E. coli* exposed to λ phages (image render dimensions: 93μm x 93μm x 15μm), (F) Final time point (t=30 h of phage exposure) of the time series acquisition in the same location as in (E). (G) Total biofilm average cell packing as a function of time following the start of phage addition into the system. The global average packing density declines with time following the addition of phages; the following panel shows that this reduction in cell packing is not homogeneous but mostly occurs along the biofilm periphery, as one would expect based on what λ phages are located. (H, F) Quantification of cell packing as a function of increasing distance from the biofilm edge toward the center of the colony in (E,F). The solid line with purple dots shows the cell packing values for the entire biofilm population, including uninfected cells, after 24 h of phage exposure. The solid yellow line shows the cell packing values for only the lysogen subpopulation, also at 24 h of phage exposure. Six hours later, the transluscent purple and yellow data points with dotted lines shown the change in cell packing a s function of distance from the colony edge. Note that the largest declines in cell packing occurs near the outer biofilm periphery, which is exactly where the lysogens are located. The drop from the solid yellow data to the light yellow/dotted line data for the lysogens shows that the biofilm cell packing around them decreases by more than 50% over the course of the 6 h time lapse due to cells undergoing the lytic cycle in proximity.

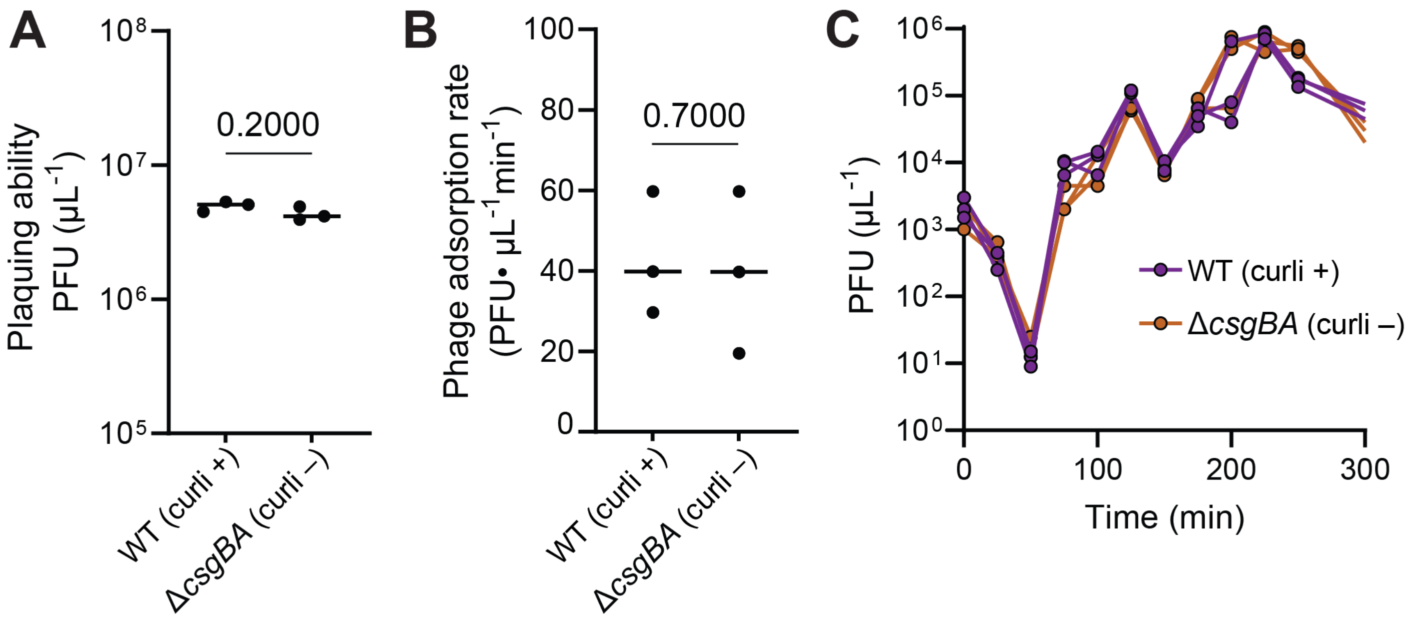

SI Figure 8. Comparison of phage susceptibility, phage adsorption rate, and overall phage population dynamics when growing with a WT *E. coli* host versus a Δ*csgBA E. coli* host. (A) The plaquing abilities of phage λ against WT (curli+) and Δ*csgBA* (curli-) cells, which are not distinguishable. (B) The adsorption rates of phage λ when co-cultured with WT *E. coli* versus Δ*csgBA E. coli*; again there is no difference. (C) The population dynamics of phage λ virions when growing with a WT *E. coli* host versus a Δ*csgBA E. coli* host. The trajectories are virtually identical, and for the brief period when trajectories are not identical (180-220) minutes, the phage titer traces for each host strain (WT versus Δ*csgBA E. coli*) are not substantially different from each other; this result indicates that the net sums of factors controlling phage titer (host susceptibility, adsorption rates, lag time, burst size) are the same for WT and Δ*csgBA E. coli*.

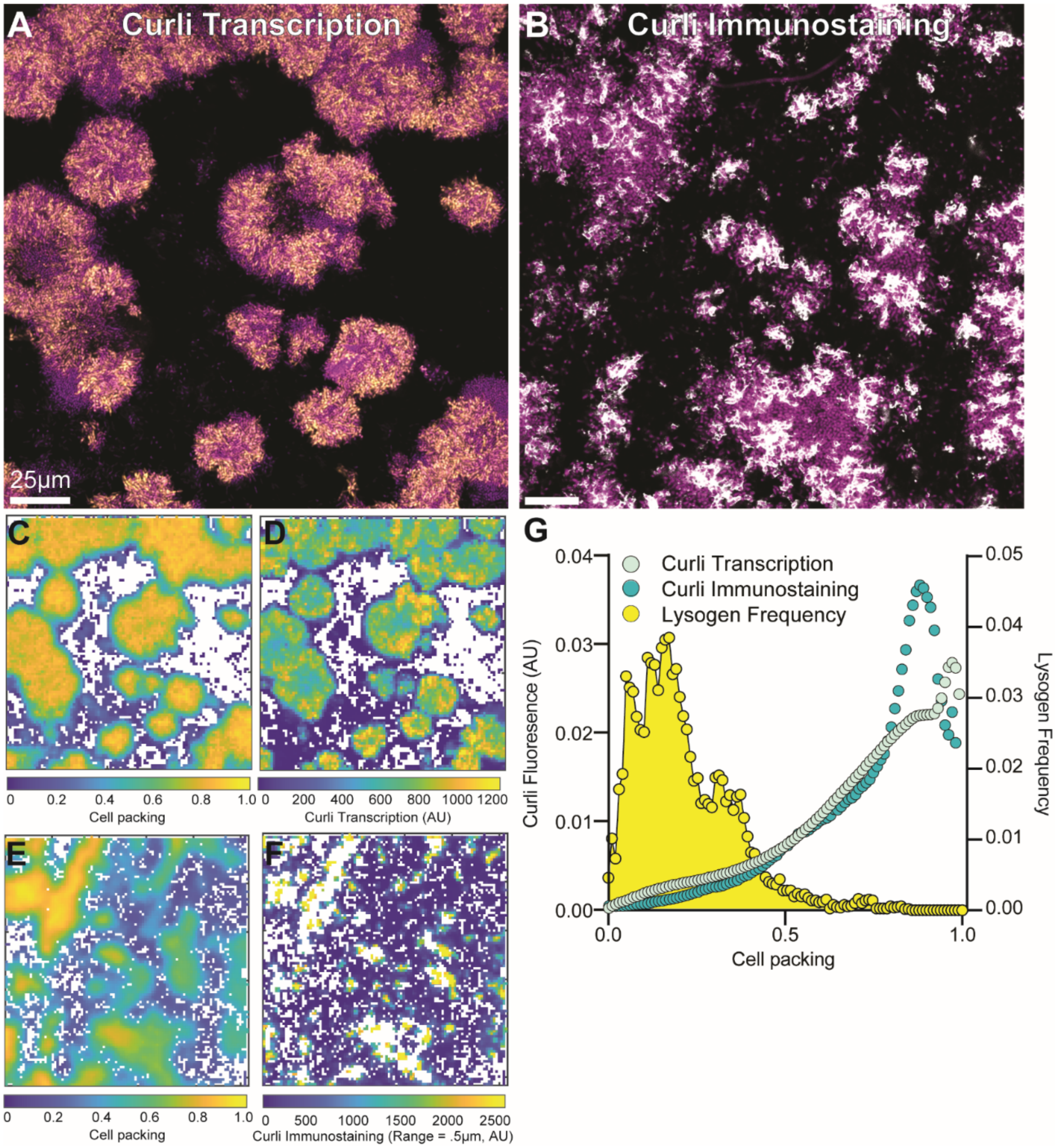

SI Figure 9. Characterization of transcriptional and translational reporters for curli production in *E. coli* biofilms grown in M9 minimal medium with 0.5% maltose. (A) Representative 2-dimensional cross section of *E. coli* (purple) containing a transcriptional reporter for the *csgBAC* operon (reporter fluorescence is shown in yellow). (B) Representative 2-dimensional cross section of *E. coli* biofilm (purple) with fluorescent immunostaining of CsgA-6xHis produced from the native genomic locus (immunofluorescence shown in white). Note that the images in (A) and (B) are 2-dimensional cross-sections to illustrate activity on the other surface and biofilm interior, but the analyses in the rest of the panels are for the full 3-D image stack capturing all cells in the field of view. (C) Heatmap of biofilm cell packing for the image in panel (A). (D) Heatmap of *csgBAC* transcription for the image in panel (A). (E) Heatmap of biofilm cell packing for the image in panel (B). (F) Heatmap of CsgA immunostaining for the image in panel (B), indicating the localization of curli matrix protein. (G) *csgBAC* transcriptional reporter and CsgA immunostaining fluorescence intensity as a function of cell packing (n=8-12). The distribution of lysogens with respect to cell packing is shown as well for comparison with the reporter signal plots; the lysogen data are recapitulated here from Figure 1 of the Main Text.

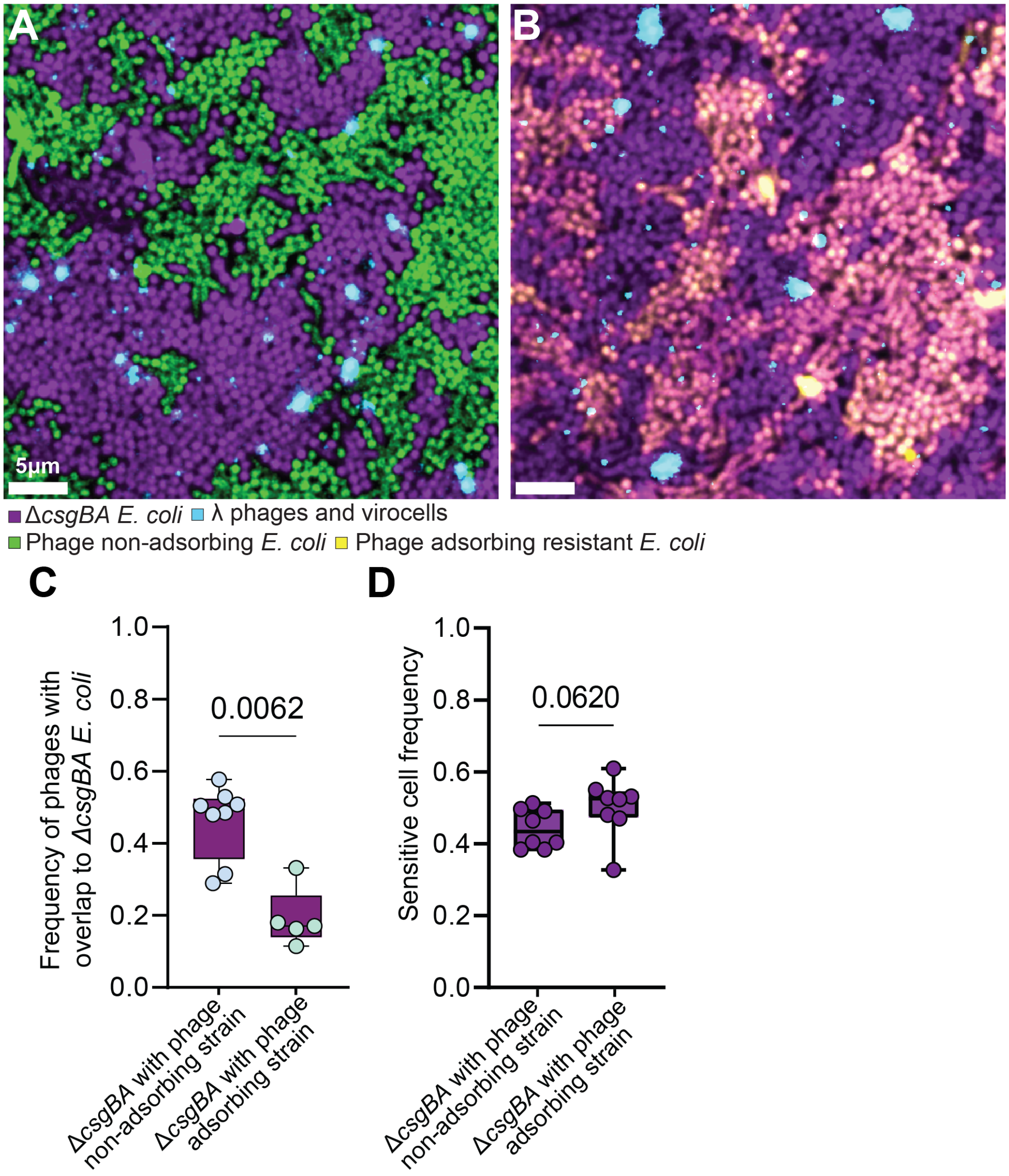

SI Figure 10. These experiments were designed to determine if phage adsorption/neutralization by lysogens could lower the exposure of nearby susceptible cells to phages. To study this question, we aimed to remove the effects of impeded phage diffusion due to biofilm architecture, and so the experiments are performed in a Δ*csgBA* curli-deficient background, which permits phage diffusion freely relative to the WT genetic background. (A) Coculture biofilms of Δ*csgBA* (purple) and Δ*csgBA*Δ*lamB* (green), inoculated with λ phages (Turquoise) for 24 h. (B) Coculture of Δ*csgBA* (purple) and Δ*csgBA* lysogens (yellow) with phage λ (cyan), inoculated with λ phages for 24 h. (C) Δ*csgBA* cells in biofilm co-culture with isogenic phage-adsorbing lysogen cells (Δ*csgBA* λ lysogen) accumulate significantly less phage fluorescence over the course of 24 h phage exposure when compared to identical experiments in which Δ*csgBA* cells were grown in co-culture with a phage non-adsorbing isogenic strain (Δ*csgBA*Δ*lamB* cells lacking the LamB receptor). This indicates that phage-adsorption by lysogens can contribute to reducing phage exposure to surrounding phage-susceptible cells. (D) A control measurement indicating that the ratio of the Δ*csgBA* strain to its co-culture strain remained close to 1:1 through the end of the experiment, to ensure that the comparison between the two treatments is not influenced by Δ*csgBA* frequency.

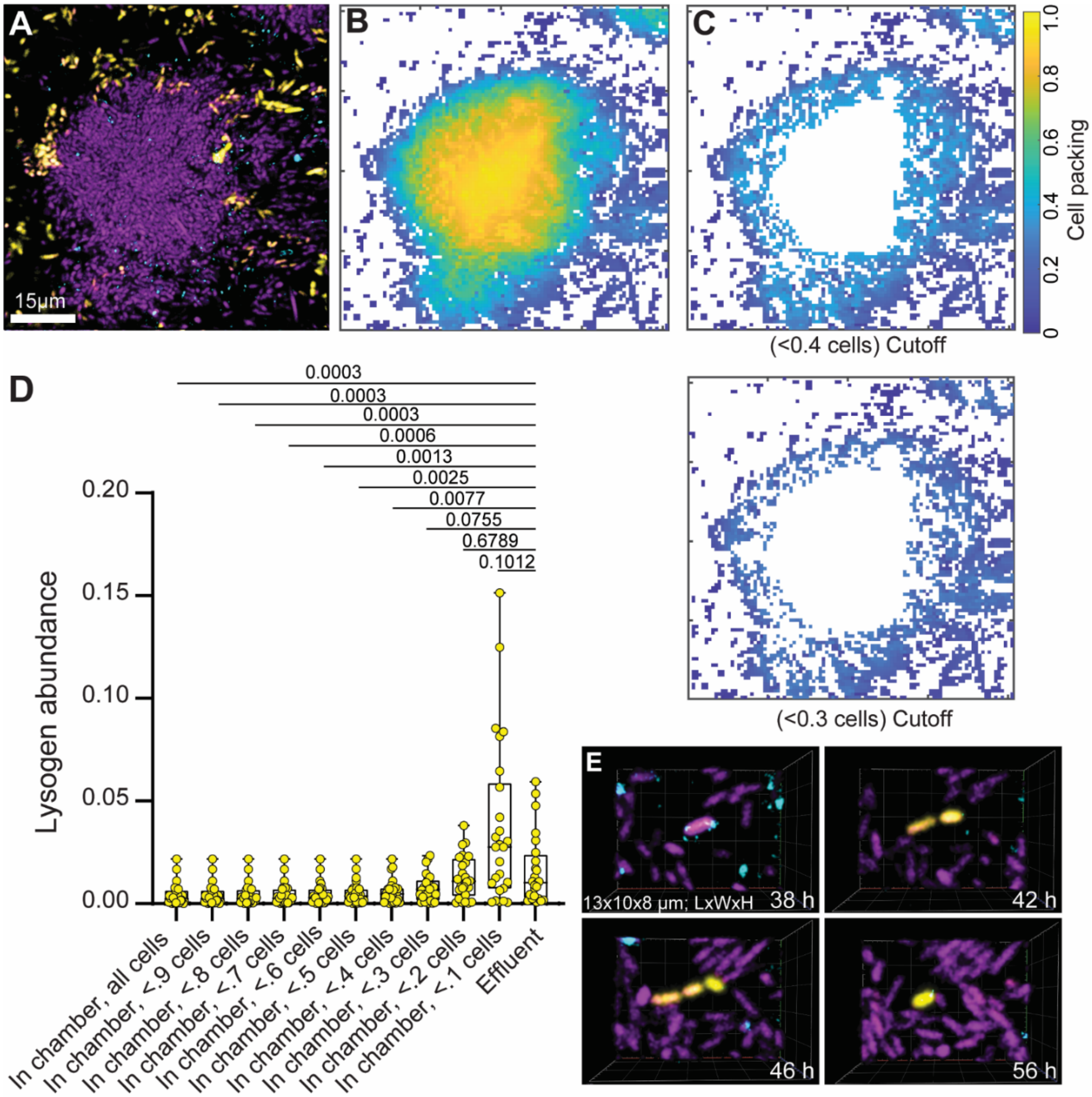

SI Figure 11. Cells leaving the biofilm chamber in the effluent are primarily derived from areas of cell packing 0.2-0.3 and lower. (A) A representative image of biofilm cluster 24 h after phage exposure. Naïve cells are purple, lysogens are yellow, and phages are cyan. (B) A heatmap of cell packing throughout the biofilm shown in panel (A). (C) An image showing only the regions of the biofilm cluster in which the cell packing fraction is less than 0.3. (D) This plot shows the frequency of lysogens in the effluent from biofilm chambers like those shown in panel A (far right bar); also shown are the average lysogen frequency in regions of the biofilm from high cell packing to low cell packing, which allowed us to find the regions of biofilm packing in which lysogen frequency is similar to that in the effluent. (E) A time series of high-magnification biofilm renderings illustrating a purple naïve host cell (purple) adsorbing λ phages (cyan), the conversion of this host cell into a lysogen (E, 42 h), whose growth and division can be observed several hours later (E, 46 h). Finally, the removal of two daughter cells is likely a cellular resolution instance of lysogen dispersal (E, 56 h). No phage induction was seen following 42 h in this experiment, so the loss of cells at this location is not due to induction but rather to removal of those cells from the biofilm altogether.

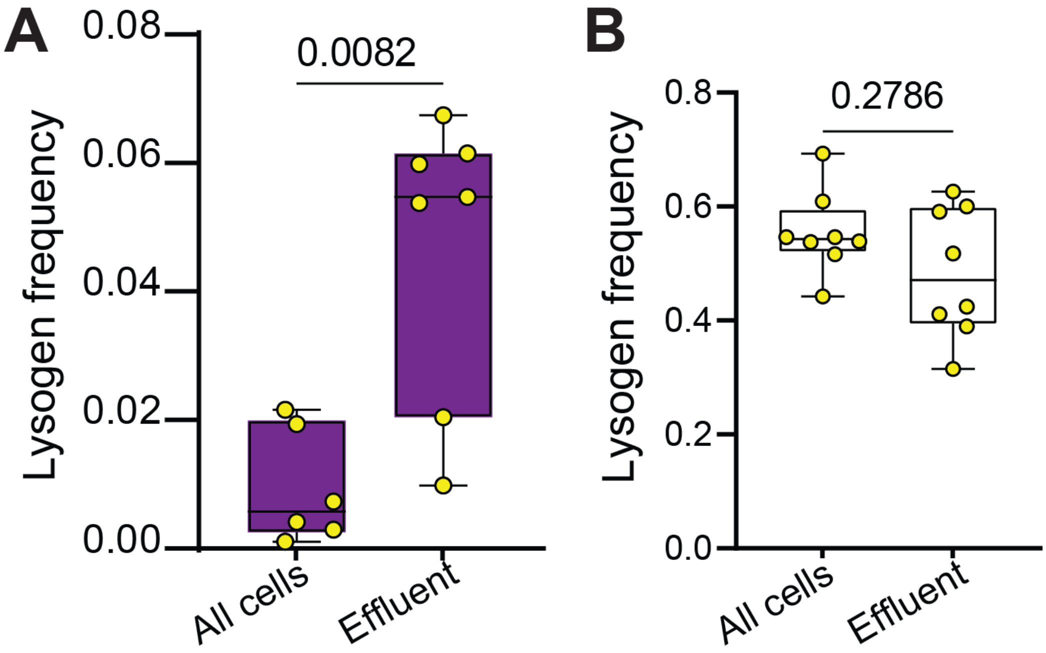

SI Figure 12. Control experiments documenting that disproportionately high lysogen dispersal persists over 72 h of phage exposure, and that lysogens and phage-naïve cells do not differ in their basic physiological disposition to disperse. (A) Biofilms of WT *E. coli* were grown for 48 h and then exposed to λ phages for 72 h prior to imagining chambers to determine the frequency of lysogens in the biofilm population, as well as measuring the frequency of lysogens in the liquid effluent of the chambers (i.e., the dispersing cells). As was the case after only 24 h of phage exposure, phages are disproportionately overrepresented in the dispersal pool relative to their frequency in the biofilm as a whole. (B) This experiment tested whether lysogens are physiologically more likely to disperse due to the introduction of the λ genome into its own. A 1:1 mixture of WT and λ lysogens were used to colonize chambers prior to biofilm growth for 48 h; no exogenous phages were added here. In biofilms co-inoculated 1:1 with WT and lysogens from the beginning of biofilm growth, the two strains remain at a ratio of ~1:1 across many experimental replicates, indicating that neither one has an inherent competitive advantage in biofilm growth over the other. Likewise, following 48 h following 1:1 inoculation of WT and lysogenized *E. coli*, the fraction of lysogens in the liquid effluent is the same as it is in the biofilm itself. This indicates that lysogens are not more physiological disposed to disperse from biofilms than WT cells. Their over-representation in the dispersal pool in the main text figures is due to the spatial arrangement of lysogens when WT biofilms are exposed to influx of phages in the liquid, *not* due to any other physiological predisposition to disperse due to the presence of the λ prophage within them.

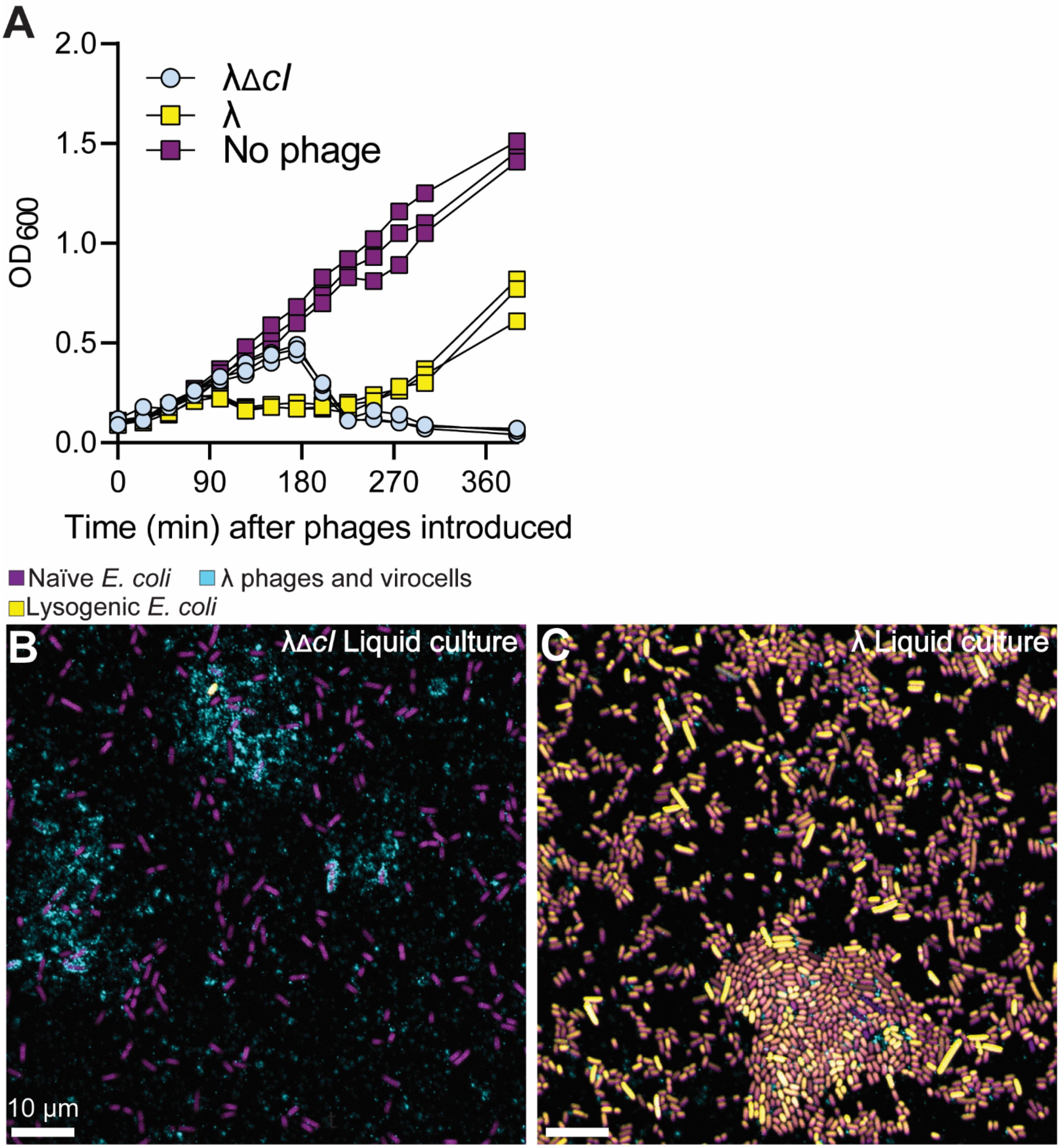

SI Figure 13. Independent shaking liquid cultures of WT cells exposed to phage λ, phage λΔ*cI*, or a no-phage control and grown at 30Cº in λ broth. After a lag period relative to the no-phage control, we observe an increase in host OD_{600} after addition of phage λ; by contrast, the host bacterial culture crashes out after addition of the obligately lytic phage derivative λΔ*cI*. This experiment verifies that the λΔ*cI* derivative behaves as an obligately lytic (virulent) phage following truncation of the locus encoding Repressor. Other than this deletion, the λΔ*cI* strain is isogenic to temperate phage λ. (B,C) Images of aliquots taken from (B) the liquid culture inoculated with WT *E. coli* and λΔ*cI* and (C) the liquid culture inoculated with WT *E. coli* and normal lysogenizing phage λ. In both cases the aliquot samples were taken at 24 h after the start of the experiment and placed on coverslips under agar pads for imaging. The images in (B) and (C) make it clear that lysogens are not created in in the *E. coli* + λΔ*cI* liquid culture, while in the *E. coli* + λ liquid culture, all cells remaining have been converted to lysogens by λ. Uninfected cells are shown in purple, lysogenized cells in yellow, and phages in cyan.

Supplemental References

1. D. O. Serra, R. Hengge, Bacterial Multicellularity: The Biology of Escherichia coli Building Large-Scale Biofilm Communities. *Annual Review of Microbiology* **75**, 269–290 (2021).

2. L. Thomason, *et al.*, Recombineering: Genetic Engineering in Bacteria Using Homologous Recombination. *Current Protocols in Molecular Biology* **78**, 1.16.1-1.16.24 (2007).

3. D. O. Serra, A. M. Richter, G. Klauck, F. Mika, R. Hengge, Microanatomy at Cellular Resolution and Spatial Order of Physiological Differentiation in a Bacterial Biofilm. *Mbio* **4**, e00103-13 (2013).

4. L. Vidakovic, P. K. Singh, R. Hartmann, C. D. Nadell, K. Drescher, Dynamic biofilm architecture confers individual and collective mechanisms of viral protection. *Nat Microbiol* **3**, 26–31 (2018).

5. J. T. Trinh, T. Székely, Q. Shao, G. Balázsi, L. Zeng, Cell fate decisions emerge as phages cooperate or compete inside their host. *Nat Commun* **8**, 14341 (2017).
